# Dissecting the Genetic Basis of Local Adaptation in Soybean

**DOI:** 10.1038/s41598-017-17342-w

**Published:** 2017-12-08

**Authors:** Nonoy B. Bandillo, Justin E. Anderson, Michael B. Kantar, Robert M. Stupar, James E. Specht, George L. Graef, Aaron J. Lorenz

**Affiliations:** 1 Department of Agronomy & Horticulture, University of Nebraska-Lincoln, Keim Hall, Lincoln, NE 68583-0915 USA; 2 Department of Molecular Genetics and Physiology of Plants, Ruhr University Bochum, Universitätsstraße 150, 40211 Bochum, Germany; 3 Department of Tropical Plant and Soil Sciences, University of Hawaii, Manoa, Honolulu, HI 96822 USA; 4 Department of Agronomy and Plant Genetics, University of Minnesota, St. Paul, MN 55108-6026 USA

## Abstract

Soybean (*Glycine max*) is the most widely grown oilseed in the world and is an important source of protein for both humans and livestock. Soybean is widely adapted to both temperate and tropical regions, but a changing climate demands a better understanding of adaptation to specific environmental conditions. Here, we explore genetic variation in a collection of 3,012 georeferenced, locally adapted landraces from a broad geographical range to help elucidate the genetic basis of local adaptation. We used geographic origin, environmental data and dense genome-wide SNP data to perform an environmental association analysis and discover loci displaying steep gradients in allele frequency across geographical distance and between landrace and modern cultivars. Our combined application of methods in environmental association mapping and detection of selection targets provide a better understanding of how geography and selection may have shaped genetic variation among soybean landraces. Moreover, we identified several important candidate genes related to drought and heat stress, and revealed important genomic regions possibly involved in the geographic divergence of soybean.

## Introduction

Soybean (*Glycine max*) is the leading legume crop produced in the world^1^, accounting for nearly half of the total world production of vegetable oils^2^ and a large majority of the oilseed meal included in livestock feed^3^. Further uses of soybean vary widely, ranging from various traditional food products such as soy milk and natto, to protein isolates included in processed foods^4^. Global soybean production has been steadily increasing for the last 50 years or more, but growth has been strongest in places far outside the original region of soybean cultivation, including diverse regions such as South America (Brazil, Argentina), northern North America (northern U.S.A, and Canada), and India^1^. This is a testament to soybeans adaptability, which will be crucially needed for agriculture in the face of a changing climate.

Following domestication from its wild progenitor *Glycine soja* (Sieb. and Zucc.) in China 3,000 to 5,000 years ago, a multitude of soybean landraces was created by ancient farmers through both artificial and natural selection^5^. These landraces were first cultivated in China for circa 3000 years before being dispersed to the neighboring regions of Japan, India, Nepal, and Russia around the first century C.E^6^. Distinct landraces emerged by the 15^th^ century C.E and ranged in areas from modern-day Japan, Korea, Vietnam, India, and Indonesia. By the 20^th^ century, it has been estimated that as many as 20,000–45,000 distinct landraces were grown by farmers in China alone^5^. Several factors, such as the self-pollinating nature of soybean and its adaptation to specific latitudinal clines due to photoperiodism led to diverged pockets of landraces that are both morphologically and genetically diverse, even from within small areas in China^5,7^. These landraces, each adapted to its specific locale and corresponding environmental conditions, have largely been replaced by modern high yielding varieties, but fortunately some of the wealth of this genetic diversity has been maintained through the storage of these landraces in germplasm banks across the world^5,8^.

The most important factor in soybean adaptation is its photoperiod response. The right combination of alleles at the “E loci”^9^, as well as the J locus controlling length of juvenility^10^, allows soybean to maximize yield and avoid frost within very narrow latitudinal ranges^5^. Beyond maturity adaptation and genetically controlled photoperiodism, information on contributors to soybean adaptation to abiotic conditions is limited, but known cases include adaptation to cold tolerance^11^, heat and drought tolerance^12^, acid soils^13^ and saline soils^14^. To our knowledge a comprehensive study on the forces shaping the genetic variation of landraces has not been performed.

Landscape genomics is a framework that aims to identify environmental factors shaping adaptive genetic variation and genetic variants that drive local adaptation^15,16^. Environmental association analysis (EAA) has emerged as a core part of landscape genomics, the goal of which is to find associations between genetic variants and environmental conditions corresponding to the locale of adaptation^15^. Studies in Arabidopsis have shown that loci implicated as being adaptive through an EAA can predict relative fitness in a common garden experiments grown across wide-ranging environments^17,18^. Recent landscape genomics and EAA studies on some of the world’s most important crops such as sorghum^19^, maize^20^, and barley^21^ provide insights into the genetic architecture and genes underlying local adaptation. No such studies have been performed in soybean, but a recent study has been performed on soybean’s wild ancestor, *Glycine soja*. Anderson *et al*.^22^ performed an EAA on 533 *G. soja* accessions and identified SNPs associated with environmental variables such as precipitation and percent sand in the subsoil. Such associations, if taken beyond this exploratory analysis and validated, could provide a useful source of variation to improve soybean given the ease in which *G. soja* can be crossed to *G. max*.

Here, we explore the genetic variation in a georeferenced collection of locally adapted landraces from a broad geographical range to help elucidate the genetic basis of local adaptation in soybean. We used geographic origin, environmental data and genome-wide SNP data to perform an environmental association analysis and discover loci displaying steep gradients in allele frequency across geographical distance and between landrace and modern cultivars. Our results provide a better understanding of how geography, environment, and selection may have shaped the distribution of genetic variation among soybean landraces, and begins to identify loci possibly associated with adaptation to diverse environmental conditions.

## Results

### Population structure, diversity, and geographical relationships among landraces

**Figure 1:**
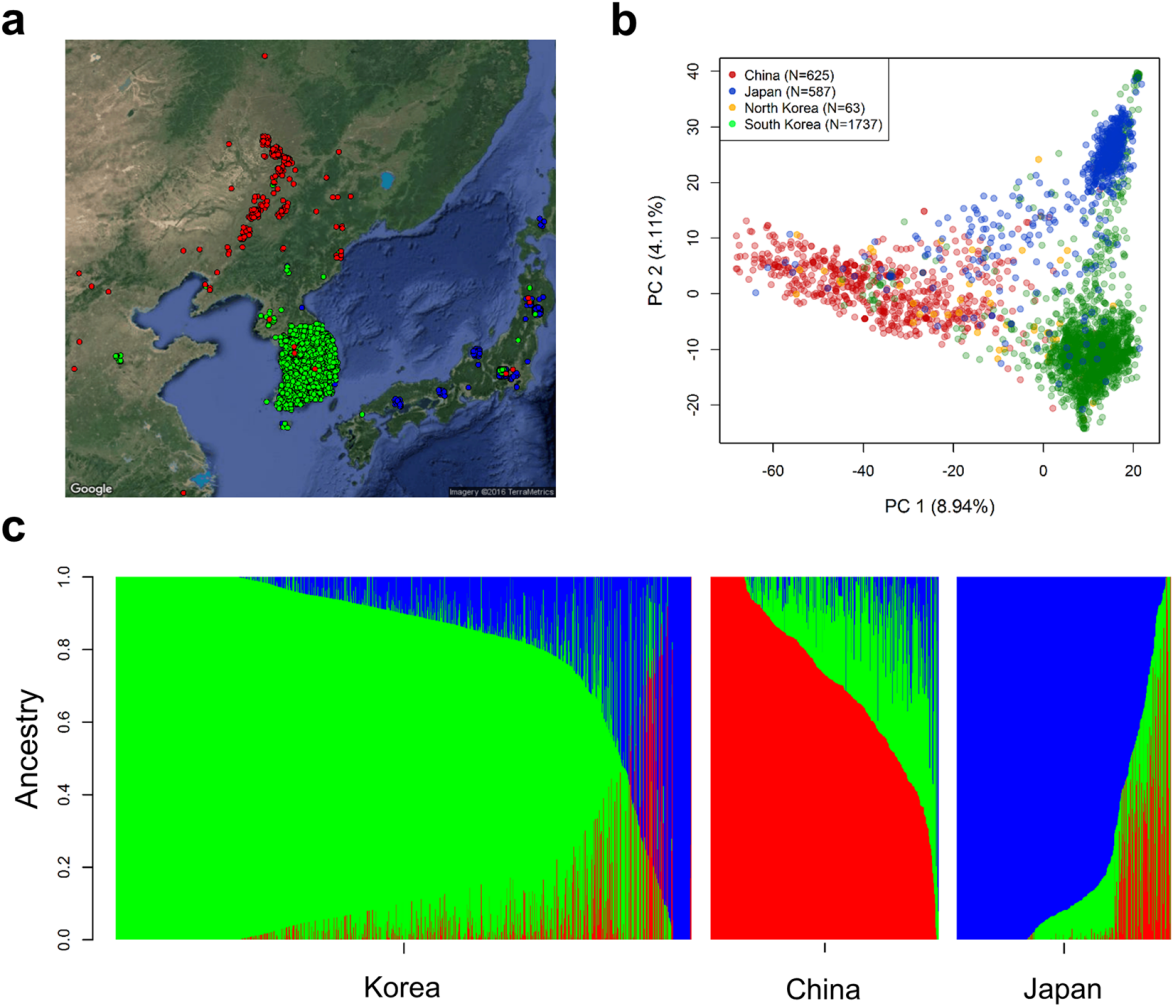
Population structure, diversity, and geographical relationships among 3,012 soybean landraces. (**a**) Results of *fast*STRUCTURE analysis in *G. max* accessions and the geographical location in which each accession was collected. The spot colors correspond to the *fast*STRUCTURE assignment (with subpopulation ancestry >80%) of each accession that generally accords with geography. The spots have been jittered to show overlapping samples. Maps were created with the *RgoogleMaps* ^54^. (**b**) Using principal component (PC) analysis of SNP data, the top two PCs which mainly accounted for geographic origin differences explained ~13% of total genetic variation. (**c**) Each colored vertical line in the barplot represents an individual accession that was assigned proportionally to the one of the three clusters. Subpopulation (SP) 1 (green cluster) represents the accessions collected from Korea; SP3 (blue cluster) forms a unique SP comprised primarily of accessions from Japan; SP2 (red cluster) is composed predominantly of accessions collected from China.

We analyzed a collection of 3,012 georeferenced soybean landraces from the geographic range of Asia (22–50°N, 113–143°E) spanning the regions of China, Japan, North Korea and South Korea (Fig. 1a; Supplementary Table S1). Genomic variation was characterized at 36,833 high quality SNPs using the SoySNP50K array^23,24^. An analysis of population structure shows subpopulation membership is largely explained by the country from which the seeds were originally collected (Fig. 1b,c). About 71% of landraces were assigned to subpopulations based on >80% ancestry, while 29% were considered to be admixed (Fig. 1c; Supplementary Table S2). After dropping admixed accessions, global genetic differentiation among countries was modest (*F*_ST_ = 0.22), with the Chinese landraces diverged from both Japanese (*F*_ST_ = 0.27) and Korean landraces (*F*_ST_ = 0.22).

**Figure 2:**
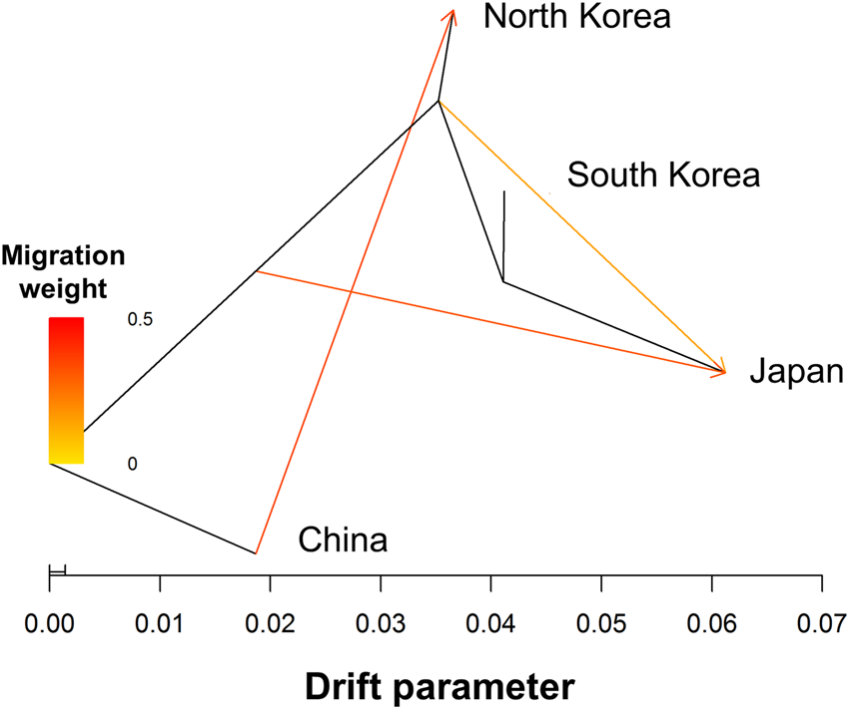
Demographic analysis on 3,012 G. max geo-reference landrace accessions. A TreeMix analysis divided the landrace population into two major geographical subdivision: the center of domestication (China) and the region of demographic expansion (Korea and Japan). The arrow corresponds to the direction of migration.

The topology of relationships^25^ determined using a TreeMix analysis on the SNP data largely recapitulates the known relationships among countries and known migration patterns in the history of soybean trade (Fig. 2). We found that estimated rates of migration were consistent with isolation by distance and amongst countries that have been in contact for a longer period of time^26,27^ (Fig. 2). Consistent with theory regarding domestication and subsequent adoption of a crop in new regions, migration occurs from the center of domestication (China) to the region of demographic expansion (Korea and Japan) (Fig. 2). While the timing of migration events is not exactly known nor estimated by this analysis, we do see a relationship between trade and the strength of migration^28^. Given the fact that these genomic data were collected with a SNP array including pre-selected SNPs, results here should be interpreted cautiously.

### Environmental variables shape the population structure of the soybean landraces

Using the passport information available from the USDA Soybean Germplasm Collection (https://npgsweb.ars-grin.gov/gringlobal/site.aspx?id=24), we compiled the biophysical/bioclimatic data for each landrace collection site, which varied widely in precipitation, temperature, soil, and altitude (Supplementary Figs 1, 2; Supplementary Table 3) (*see Methods*). Sites within China are generally colder and drier (i.e., continental climate) compared to Korea and Japan (more maritime climate) (Supplementary Fig. 2). During the soybean growing season in this region (March-November), precipitation and temperature were positively correlated (Supplementary Figs 3, 4). A principle component (PC) analysis revealed extensive multicollinearity among environmental variables, with the first four PCs explaining ~86% of the total variation (Supplementary Fig. 5a). Plotting the first two PCs largely recapitulates the geographical distribution of collection sites (Supplementary Fig. 5b).

**Figure 3:**
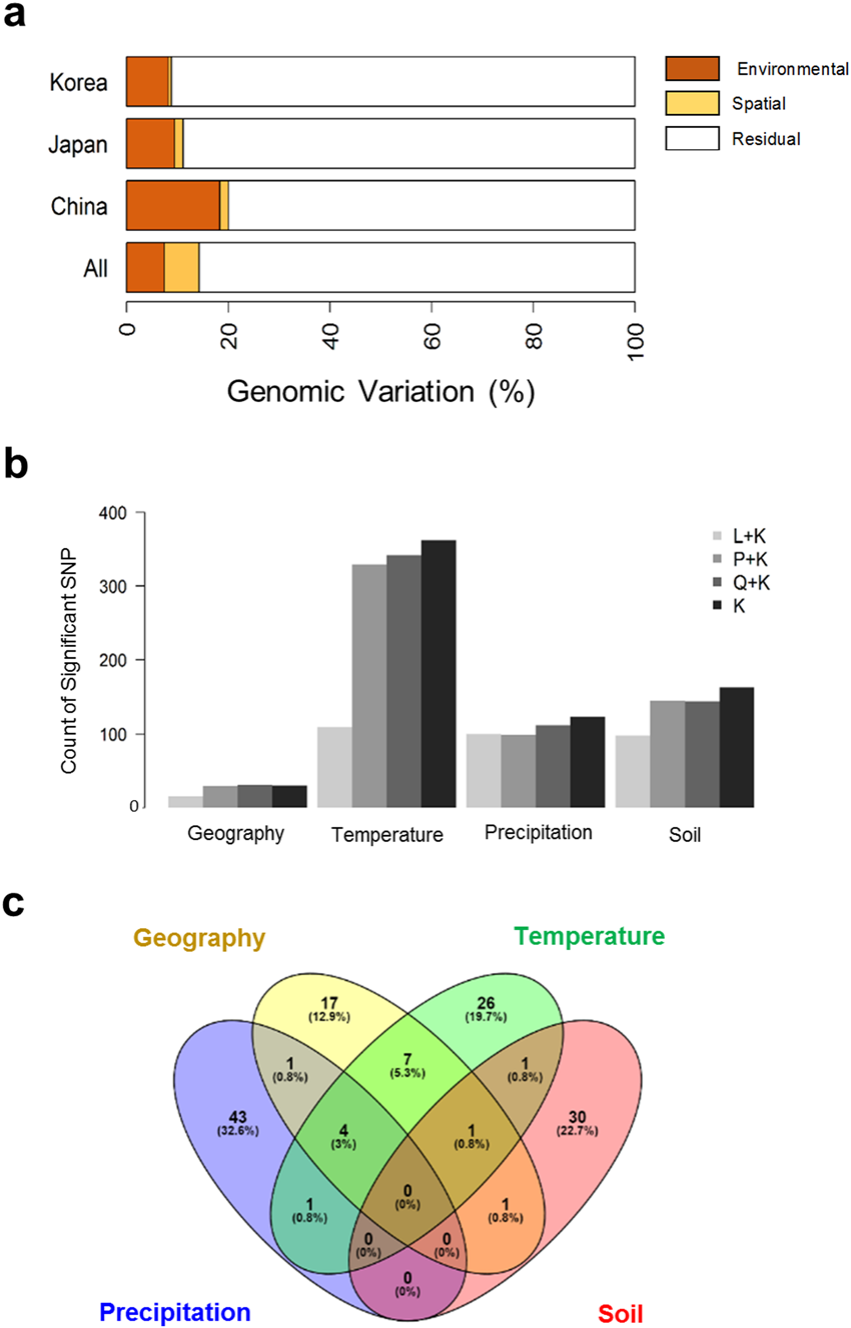
Environmental association analysis on 3,012 G. max geo-reference landrace accessions. (**a**) Partitioning of genomic variation due to environmental variation using a partial redundancy analysis. Genomic variation was partitioned based on four categories of grouped environmental variables (spatial, temperature, precipitation, soil). (**b**) Summary of genome-wide significant associations identified using four linear mixed models. (**c**) Summary of unique and overlapped significant associations in four categories of environmental variables (spatial, temperature, precipitation, soil).

We aimed to determine how environmental factors have shaped genetic diversity among soybean landraces. Because soybean adapts to latitudinal zones, largely via genetic alteration of photoperiodism, genomic variation is likely to be strongly correlated with latitude. To condition the effects of spatial variation, we performed a partial redundancy analysis^29^ to find linear combinations of SNPs using linear combinations of environmental variables such that the SNP variation explained was maximized. After accounting for variation in latitude and longitude, temperature variables explained a larger portion of genomic variation than variables related to soil and precipitation (5.2% versus 3.4 and 3.3%) (Fig. 3a). Altogether, environmental and spatial variables cumulatively explained 14.3% of the genomic variation; 6.9% was explained by spatial variation (Fig. 3a). Genomic variation explained by environmental variables was found to be greater in the region of domestication (China = 18.35%) than in the regions of demographic expansion (Korea = 8.25% and Japan = 9.43%) (Figs 2; 3a). Our results show that spatial and temperature variables played an important role in shaping the existing genetic variation. A Mantel’s test supported this finding, indicating that isolation by latitudinal variation influences genetic divergence in the landrace population (*r* = 0.579, *p* < 0.0001).

### Landscape genomics provide insights into genetic architecture of local adaptation

**Figure 4:**
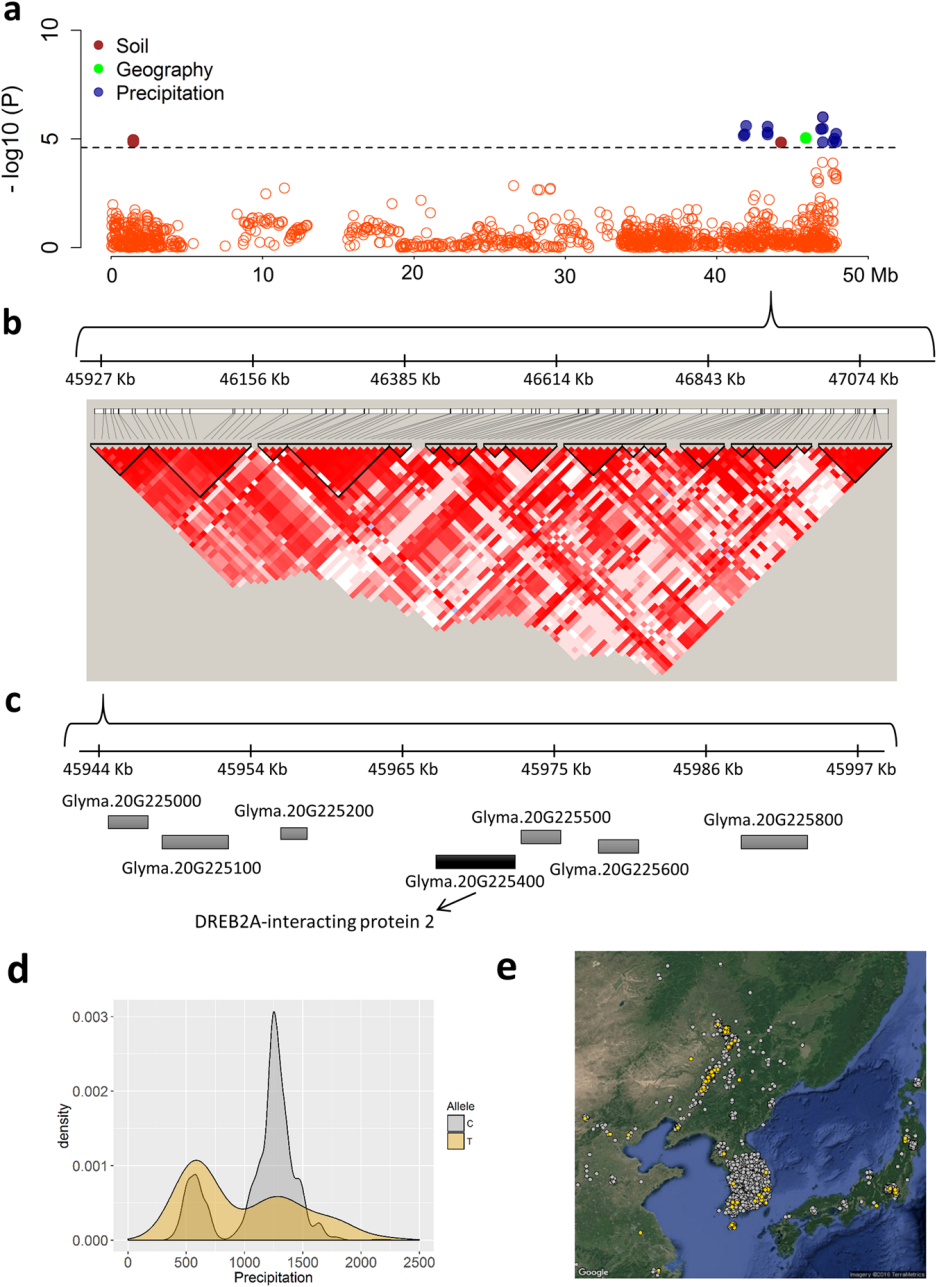
Environmental associations identified on chromosome 20 of the *Glycine max* genome. (**a**) Significant association signals were identified for geography, soil and precipitation variables between 45864382–47884469 bp on chromosome 20. (**b**) Linkage disequilibrium and haplotype analysis using the four gamete algorithm within the range of significant SNP between 45864382–47884469 bp. (**c**) Zoomed-in view of a narrowed region around the significant markers. The Arabidopsis ortholog for the nearest gene, Glyma.20G225400, is annotated as Dehydration-Responsive Element Binding Protein2a (DREB2A). (**d**) Density plot of allele frequency distribution for annual mean precipitation. (**e**) Geographic location of individuals with the C allele (gray) or T allele (gold) with jitter added to show overlapping samples. Maps were created with the *RgoogleMaps* ^54^.

**Figure 5:**
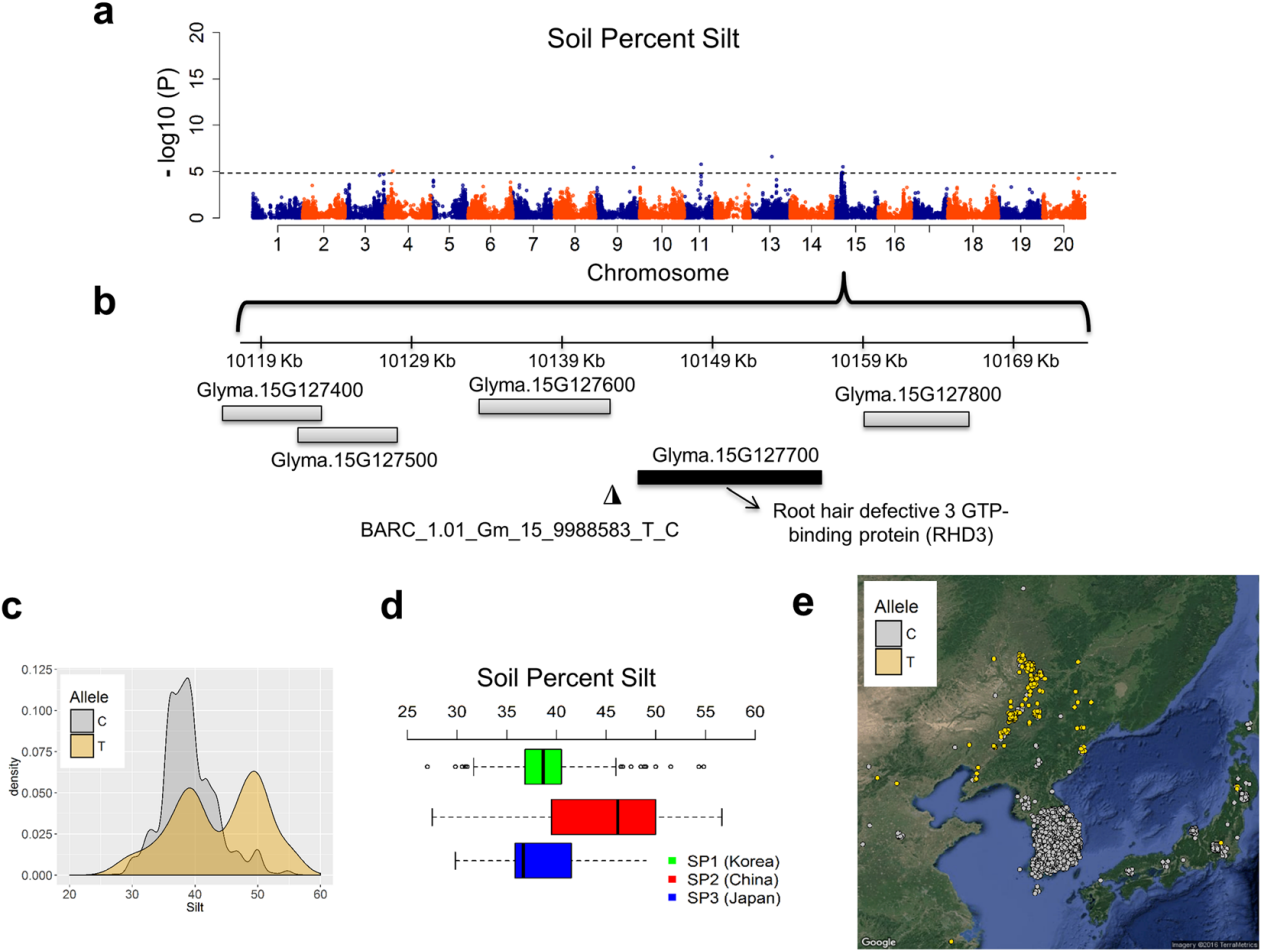
Genome-wide environmental association results for soil percent silt. (**a**) Genome-wide view of association results for soil percent silt. A cluster of significant associations was identified on chromosome 15 across six soil depths. (**b**) Zoomed-in view of a 50 kb region around the significant marker BARC_1.01_Gm15_9988583_T_C, the most significant hit for soil percent silt. The Arabidopsis ortholog for the nearest gene, Glyma.15g127700, is Root Hair Defective 3 GTP-binding protein (RHD3). (**c**) Density plot of allele frequency distribution for Percent Silt. (**d**) Boxplot analysis indicates substantial variation among subpopulations for soil percent silt. (**e**) Geographic location of individuals with the “C” allele (gray) or “T” allele (gold) with jitter added to show overlapping samples. Maps were created with the *RgoogleMaps* ^54^.

We performed an environmental association analysis^15^ to identify candidate loci that could contribute to local adaptation. Four mixed linear models were fitted to correct for confounding effects due to population structure (Fig. 3b; Supplementary Tables 4–8). The Q + K model was chosen for reporting of associated loci because it sufficiently reduced false positives compared to the other models; we expected it to not eliminate too many associations with environmental variables correlated with latitude as we would expect when using the L + K model (Fig. 3b; Supplementary Fig. 6). A total of 73 distinct genomic regions were identified across 78 environmental and spatial variables (Supplementary Table 8). We found substantial overlap in associated loci within and between variable categories (Fig. 3c). The non-overlapping SNPs between traits were highest for precipitation (32.6%), followed by soil (22.7%), temperature (19.7%) and spatial variables (12.9%) (Fig. 3c). About 37% of associated loci were consistently identified in monthly and seasonal/annual variables which was likely a result of these variables being strongly correlated with one another, although pleiotropy could play a role (Supplementary Fig. 4). SNPs associated with environmental variables tended to cluster together with the most notable region being on chromosome 20, which showed a long range of associated SNPs between 45864382–47884469 bp (Fig. 4a). This region is characterized by long-range LD and contains candidate genes associated with drought and cold tolerance such as DREB2A^30^ (Fig. 4b,c). Allelic effects of the closest SNP tagging DREB2A indicate that the C allele is associated with high precipitation environments while the T allele is associated with low precipitation environments (Fig. 4d,e). We also found a novel genomic region on chromosome 15 (9840775–10142301 bp) that includes important candidate genes for soil adaptation. This region associated with soil silt content is 3.63 kb away from Glyma.15G127700 which encodes for Root Hair Defective 3 GTP-binding protein (*RHD3*), required for appropriate root growth^31^ (Fig. 5a,b). Allelic effect estimates (Fig. 5c) and geographic distributions (Fig. 5d,e) indicate that accessions carrying the T allele tend to thrive in soil with higher silt content. Overall, many small-effect variants that cumulatively explained ~10% of phenotypic variation were identified. The locus of largest effect only explained ~5% of the total variation for mean precipitation in the wettest quarter of the year. The low value is not unexpected given that environmental and spatial variables cumulatively explained only 14.3% of total genomic variation (Fig. 3a).

**Figure 6:**
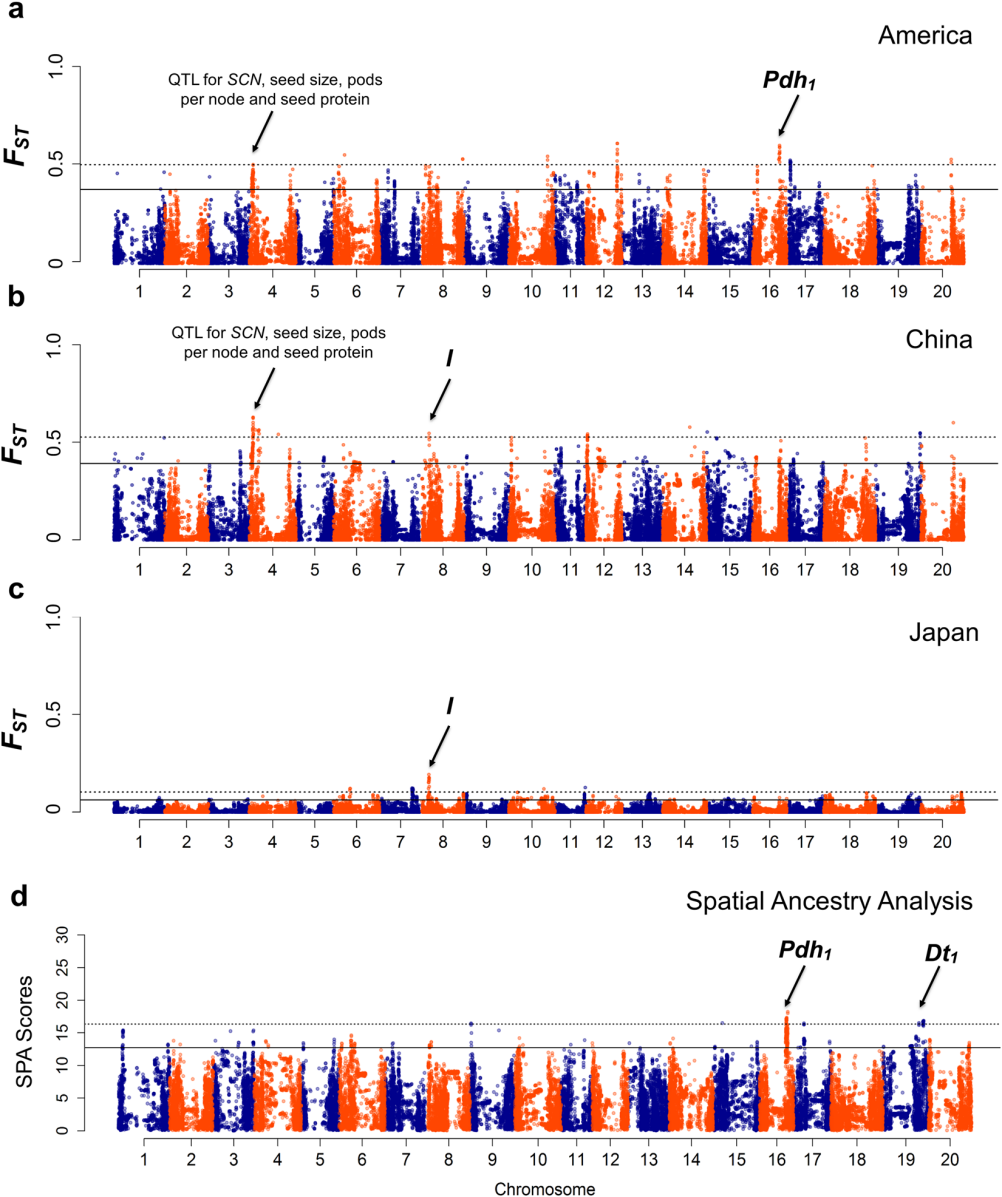
Identification of putative loci underlying selection using *F*_*ST*_ and Spatial Ancestry Analysis (SPA). *F*_*ST*_ analysis between elite and landrace population within each country: (**a**) America, (**b**) China, and (**c**) Japan. The *F*_*ST*_ values are plotted against the base pair position on 20 chromosomes of soybean. The dashed horizontal line denotes the calculated *F*_*ST*_ value based based on 99.9^th^ percentile for declaring a selected region. The solid horizontal line denotes the calculated *F*_*ST*_ value based on 99^th^ percentile for declaring a selected region. Strong selection signals that co-localized with known genes or QTL are indicated by an arrow. (**d**) SPA of 3,012 geo-referenced landrace accessions in *G. max*. The SPA selection scores are plotted against the base pair position on 20 chromosomes of soybean. Strong selection signals that co-localized with known genes or QTL are indicated by an arrow. The dashed horizontal line denotes the calculated SPA threshold score based on 99.9^th^ percentile for declaring a selected region. The solid horizontal line denotes the calculated SPA threshold score based on 99^th^ percentile for declaring a selected region.

Genomic regions harboring signatures of selection that are specific to a country of origin could reflect adaptation to local agricultural practices. We performed an *F*_*ST*_ analysis between soybean accessions identified as cultivars and landrace populations for each country to identify possible targets of selection specific to a country (Supplementary Table 9). A total of 24 genomic regions (China = 11; Japan = 5; North America = 8) were identified (Fig. 6; Supplementary Fig. 7a,b). The strongest target of selection in Japan (Fig. 6c) was associated with development of specialized products (i.e, clear hilum color) while the strongest target of selection found within China (Fig. 6a) and America (Fig. 6b) were associated with genetic improvement for yield and disease resistance (e.g., shattering resistance, SCN) (Supplementary Fig. 7a,b). Nearly all selected regions within country were distinctive with only two genomic regions being common between countries (Fig. 6). An overlapping region on chromosome 4 (4384695 bp) is a reported QTL hotspot for important agronomic traits^32–34^ (Fig. 6a,b). The second overlapped region on chromosome 8 (8451046–8602715 bp) co-localized with the *I* locus (selected in China and Japan; Fig. 6b,c), which controls the distribution of anthocyanin pigments for pod and hilum color^35,36^.

To complement the *F*_*ST*_ results, we identified loci showing steep gradients in allele frequency in the 3,012 geo-referenced landrace accessions using Spatial Ancestry Analysis (SPA)^37^, which gives a comparable measure where high values are consistent with selection for adaptation (Fig. 6d). Using an outlier test to identify the SNPs ranking in top 0.1%, six strongly selected genomic regions were identified, including genes influencing stem termination *(Dt1* ^38^) and shattering resistance *(Pdh1* ^39^) (Fig. 6d).

**Figure 7:**
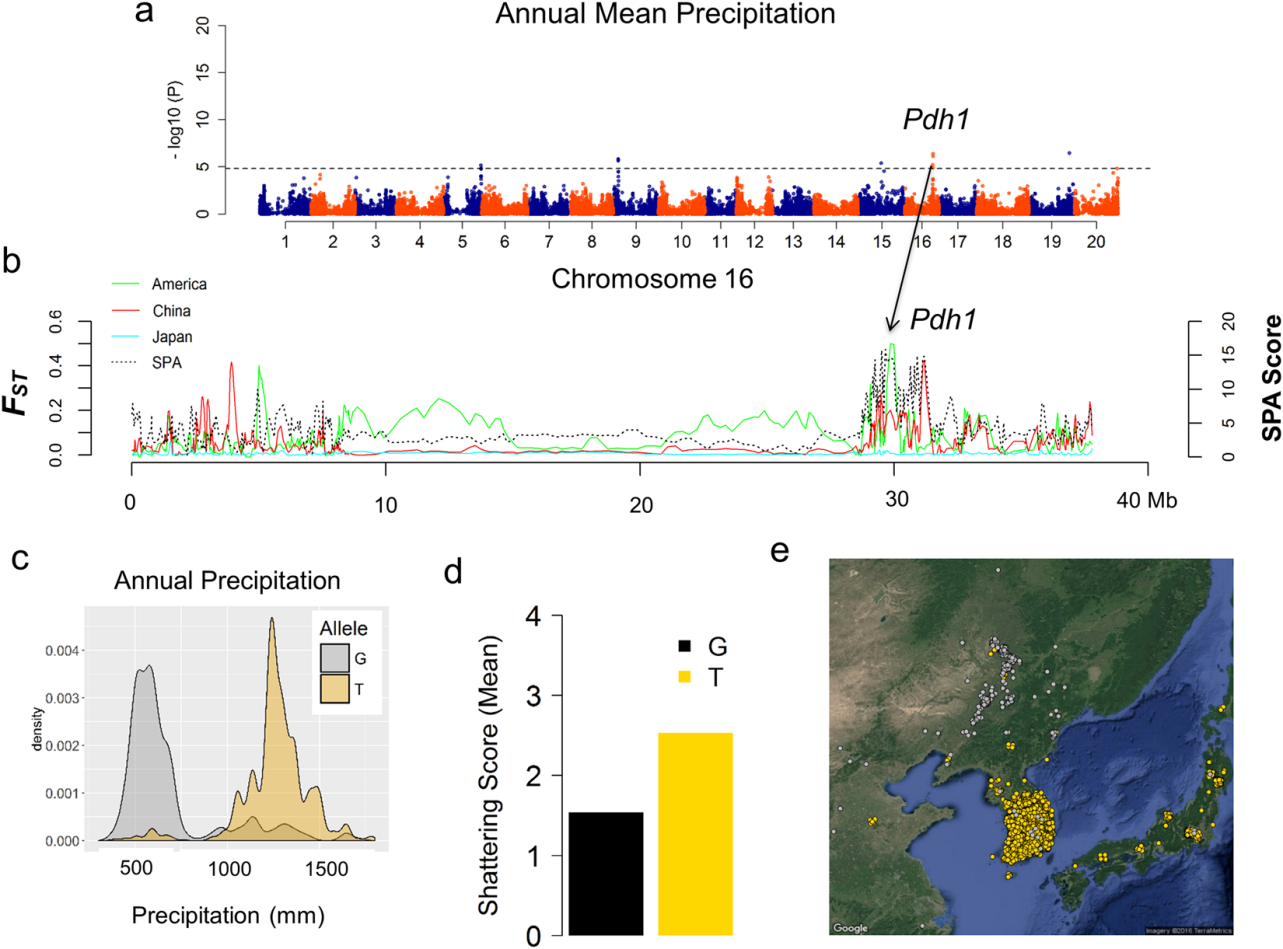
Environmental association, spatial ancestry analysis (SPA) and *F*_ST_ identified associated SNP that co-localized with *Pdh1* on chromosome 16. (**a**) Environmental association for Annual Mean Precipitation identified significant SNP associations between 29517407–31181902 bp that co-localized with *Pdh1*, a major QTL responsible for the reduction of pod shattering in soybean. (**b**) SPA and *F*_ST_ values were plotted based on a sliding-window approach. Notably, highest SPA and *F*_ST_ values overlapped with *pdh1*. (**c**) Density plot of allele frequency distribution for Annual Mean Precipitation. (**d**) Allelic effects of the strongest associated SNPs for shattering scores (mean). (**e**) Geographic location of individuals with the “G” allele (gray) or “T” allele (gold) with jitter added to show overlapping samples. Maps were created with the *RgoogleMaps* ^54^.

Finally, we aimed to identify loci that are strong candidates for contributing to adaptive variation by identifying those that were found both by our environmental association analysis and F_*ST*_/SPA analysis. Five associated regions of overlap between environmental association, *F*_ST_ and SPA were found (Supplementary Fig. 7a,b), including the two widely known genes, *Dt1* and *Pdh1*, which were both associated with precipitation and temperature variables (Fig. 7; Supplementary Fig. 9). Examining allelic effects of the SNP with the strongest association indicated that *Dt1* and *Pdh1* are more strongly associated with precipitation than temperature (Supplementary Fig. 9). *Dt1Dt1* genotypes are indeterminate (i.e., main stem tip remains vegetative despite floral induction) while *dt1dt*1 genotypes are determinate (i.e., main stem tip becomes an inflorescence meristem and a terminal flower upon floral induction). Indeterminate genotypes, mostly from China, appear to be more adapted to drier climates (Supplementary Figs 8; 9a,c) possibly because their vegetative and reproductive periods overlap. Determinate accessions, predominated in Japan and Korea (Supplementary Figs 8; 9a,c), have shorter flowering periods (beginning of bloom to full bloom) which could make them vulnerable to drought events^40,41^.

Variants surrounding the *Pdh1* locus exhibited high F_*ST*_ values as well as strong associations with precipitation variables (Fig. 7a–c), highlighting its importance to soybean adaptation. The SNP T allele appears to be linked to *Pdh1* as landraces carrying this allele tend have higher shattering scores. (Fig. 7d). Our results suggest that stronger selection for *pdh1pdh1* genotypes was imposed in areas of low precipitation as more arid conditions exacerbates shattering (Fig. 7d,e). Alternatively, *Pdh1Pdh1* genotypes could have been tolerated in Japan and Korea due to a humid climate and thus minimal shattering stimulus, thereby allowing pods to mature and be collected before shattering would preclude a successful gathering of the plants.

Three additional genomic regions were both targets of selection and were found to be associated with environmental variables, making them strong candidates for harboring genes important to adaptation to local conditions. A chromosome 9 region (Supplementary Fig. 10) co-localized with a Ca^2+^-dependent lipid-binding protein (AtCLB) and 2OG-Fe (II) oxygenase family protein (Supplementary Fig. 10b). The AtCLB confers an enhanced drought and salt tolerance in *A. thaliana* ^42^. The 2OG-Fe (II) oxygenase is important for early iron deficiency chlorosis signaling in soybean^43^ and is associated with resistance to downy mildew^44^. A chromosome 17 region associated with longitude, soil and temperature variables (Supplementary Fig. 11) contained a heat-shock transcription factor and calmodulin-binding factor which is a homolog of *CAMTA1* related to drought responses in *A*. thaliana^45^. Calmodulin-related Calcium sensor plays a role in trichome branching which protect plants from abiotic stresses^46^.

## Discussion

The United States produces around 40% of the world’s soybeans, with production being concentrated in the upper Midwest region^47^. Climate change has been shown to adversely affect soybean production in the USA. Mourtzinis *et al*.^48^ reported that potential yield gains in soybean from 1994 to 2013 were suppressed by as much 30%, possibly resulting in an $11 billion loss. During the 33-yr time frame between 1979 and 2011, higher summer temperature have become the norm, contributing to a remarkable geographical shift in the location of soybean production which has now become more concentrated in the Upper Midwestern United States^47^. Development of soybean varieties better adapted to changing environments and local conditions could help to ameliorate negative effects due to climate change.

Environmental association analysis is a powerful tool to identify genes contributing to adaption to local environmental conditions. The landrace collection within the USDA Soybean Germplasm Collection contains a wealth of genetic diversity adapted to wide ranging environmental conditions. Through the application of an EAA to this collection, we found that both environmental and spatial variables have shaped the genetic diversity of soybean landraces. Among the environmental variables, temperature variables explained the most genomic variation, which agrees with the fact that increasing temperature has depressed soybean yields more than changing precipitation patterns^48^.

In addition to helping to reveal the predominant forces shaping adaptation, our EAA identified many specific loci putatively contributing to local adapation in soybean. All of these loci had small effects, cumulatively explaining only at most 10% of the variation in an environmental variable. The largest effect locus explained only 5% of the variation in mean precipitation of the wettest quarter. These findings suggest that, unsurprisingly, the genetics underlying adaptation to environmental conditions are highly complex. However, we cannot fully and conclusively answer such questions because (1) the weather data stored in the WorldClim database does not necessarily perfectly represent the weather conditions during the time of soybean landrace adaptation, and (2) the geographical coordinates associated with each landrace held in the Collection likely do not represent the exact original location each landrace was adapted to.

We did find that the well-known loci of *Pdh1* and *Dt1* had divergent allele frequencies across locations with differing temperature and precipitation profiles. While it is expected that landraces with different shattering and stem termination phenotypes would be found in different environments, this is the first time this has been quantified and demonstrated using genomic, environmental, and phenotypic data. This observation helps to reinforce the validity of our associations on other, yet-to-be characterized loci.

Overall, 73 distinct genomic regions were identified across 78 environmental and spatial variables, nearly 40% of which were associated with at least two environmental variables. We noted important abiotic-stress responsive genes very near or directly underneath SNP associations. For example, *DREB*2*A*, *AtCLB*, and *CAMTA1* are all important genes related to drought and cold tolerance. Notably, we found a putative novel genomic region for soil adaptation which encodes for *RHD3* that plays an important role for root growth and development^31^. These narrowed candidate genes are essential to achieve a better understanding of drought and heat tolerance in soybean.

Increasing soybean productivity will require adapting the crop to new locations and changing weather patterns. The results of this study can assist soybean breeders in identifying germplasm accessions that can be used as donor parents for breeding soybean cultivars for a changing climate. This SNP-level knowledge on adaptation could also be used as prior information for genomic prediction of yield under specific environmental conditions^19^. Future common garden experiments should also be conducted to help validate associations, followed by confirmation through gene editing. This research framework could greatly contribute to our understanding of soybean adaptation and eventually help to discover and extract useful alleles from germplasm collections.

## Materials and Methods

### Plant Materials

#### Landrace Collection for Environmental Association and Spatial Ancestry Analysis

The set of landrace accessions used in this study are from the USDA Soybean Germplasm Collection. Only lines with latitude and longitude coordinates were included. These were a subset of the 5,396 accessions previously labeled as landraces^24^, or *G. max* lines added to the USDA collection prior 1945 sourced from China, Japan, North Korea, and South Korea. This threshold was meant to eliminate elite lines developed through modern breeding practices. We then omitted those accessions determined to be genotypic duplicates and accessions that were potential geographic outliers. Filtering left a total of 3,012 landrace accessions that were collected within the geographic range of 22–50°N and 113–143°E. Landrace accessions were distributed in China (N = 625), Japan (N = 587), South Korea (N = 1,737) and North Korea (N = 63) (Supplementary Table 1).

### Elite and Landrace Collections for analysis of F_ST_

Plant materials for selection mapping were comprised of landrace and elite populations recently described^24^. As our objective was to identify genomic regions that were subject to selection for local adaptation, we partitioned elite and landrace collections based on country of origin Supplementary Table 9. China had the highest proportion of landrace accessions (N = 2,727), followed by South Korea (1,776), and Japan (N = 893)^24^. As no landrace accessions originate from North America, we chose the known ancestors of North American soybean^49^ to identify targets of selection. A total of 65 *G. max* landrace accessions were extracted for North America, all introduced from Asia^24,49^. The breeding programs of Japan, China and North America have produced a large number of modern cultivars^5^. In this study, the set of modern cultivars was comprised of 565 North American cultivars, 364 cultivars from China, 615 cultivars from Japan and 25 cultivars from Korea (Supplementary Table 9). These were independent from the landrace accessions. We omitted the Korean population for the F_*ST*_ analysis because of the small population size for Korean elite lines which may confound the selection mapping results.

### Genotype Data

Genotype data from the SoySNP50K platform were downloaded from SoyBase (Grant *et al*. 2010) for all available *G. max* landrace and elite accessions^24^. Ambiguous and heterozygous SNP calls were treated as missing data due to the low outcrossing rate in *G. max* ^5^. The physical map positions of the SoySNP50K SNPs^23^ were mapped into the second genome assembly ‘Glyma.Wm82.a2’^22^. Any SNP with minor allele frequency (MAF) <0.01 was removed from the genotype dataset for subsequent analyses. The SNP genotype data set is publicly available at http://www.soybase.org/dlpages/index.php.

### Environmental Data

#### Climate Data

The latitude and longitude coordinates of 3,012 *G. max* accessions were used to query the WorldClim database (see http://www.worldclim.org/) for 67 environmental variables (Supplementary Fig. 1), including bioclimatic variables based on yearly, quarterly, and monthly temperature and precipitation data as well as altitude data at a resolution of 30 arc-seconds (approximately 1 km grids)^50^ (Supplementary Table 3). The bioclimatic variables represent annual trends, seasonality and extreme or limiting environmental factors that are often used in ecological niche modeling (Hijmans *et al*.^50^). The unit used for downloaded temperature data are in °C * 10. This means that a value of 231 represents 23.1 °C. Temperature data was converted into °C by dividing the temperature value by 10. The unit used for the precipitation data is millimeter (mm).

#### Soil Data

The sampling locations of 3,012 landrace accessions were also used to query the ISRIC database^51^ (World Soil Information database) for seven biophysical variables (pH × 10 in H_2_O, percent sand, percent silt, percent clay, bulk density in kg/m^3^, cation exchange capacity in cmolc/kg, and organic carbon content (fine earth fraction) in permilles) at a resolution of 30 arc-seconds (Supplementary Table 3) (see http://www.isric.org/). Available data for seven biophysical variables were taken at six soil depths: 2.5 cm, 10 cm, 22.5 cm, 45 cm, 80 cm, and 150 cm^51^. Because of high correlation and less variability in soil variables across depths, we grouped the six measurements per variable into one class by taking the average value across soil depths, leaving us with seven soil variables.

Principle component analysis on the bioclimatic and biophysical variables (first scaled to a mean of 0 and standard deviation of 1) was conducted using the prcomp function in R. Pearson correlation coefficients between bioclimatic and biophysical variables were calculated in R. Boxplots for each scaled bioclimatic and biophysical variable were created based on *G. max* localities to examine the distribution for each variable (Supplementary Fig. 2).

### Population Structure and Linkage Disequilibrium

Principal component analysis using SNPs present in all landrace accessions was conducted using the *prcomp* function in R. The Bayesian clustering program *fast*STRUCTURE^52^ was used to calculate varying levels of K (2–10) and the command chooseK.py was used to identify the model complexity that maximized the marginal likelihood (K = 2–6). The population structure was visualized using barplot based-function in R. Genome-wide and intra-chromosomal linkage disequilibrium (LD) were estimated using pairwise *r* ^*2*^ between SNPs, which was calculated using PLINK version 1.07^53^. All geographical maps in this study were created with the *RgoogleMaps* ^54^ package in R (https://cran.r-project.org/web/packages/RgoogleMaps/RgoogleMaps.pdf).

### Partitioning of Genomic Variation

We calculated the proportion of genome-wide SNP variation explained by environmental variables. We used variance partitioning of redundancy analysis (RDA) implemented in the R package *vegan*. The RDA is an eigenanalysis ordination to assess the explanatory power of multivariate predictors (environmental and geographical variables) for multivariate responses^29^ (e.g., SNP data). The variance components explained by environmental variables were partitioned by fitting different models. The first model considered all environmental and geographic variables as explanatory variables and the SNP data as response variables. Because geographic effects are correlated with the SNP data, we fit a partial model in which the SNP data were conditioned on the effects of geographic coordinates. For both models, significance testing was conducted using Monte Carlo permutations test with 500 runs and α = 0.01.

### Identifying Targets of Selection with FST and Spatial Ancestry Analysis

*F*_*ST*_ outlier analyses and Spatial Ancestry Analysis (SPA) were used to identify loci with differential allele frequency across groups or geographical space, which could be caused by selection. To identify loci that had been selected locally, *F*_*ST*_ analysis was conducted between elite and landrace populations within each country (*F*_*ST within*_). Theta (Ѳ), the variance-based *F*_ST_ estimate^55^ was estimated using the R *hierfstat* package^56^. For visualization, *F*_ST_ was averaged in sliding windows, with a window size of five and a step of 3 SNPs^22^. SNPs with *F*_*ST*_ values above the 99.9^th^ percentile were identified as outliers. A Mantel test was conducted to explore isolation by distance utilizing great circle distance between geographic locations and pairwise genetic distance using the *vegan* package in R.

SPA was used to detect loci showing steep gradients in allele frequency^37^. The SPA incorporates geographic and genetic gradients in identifying local clines. This type of analysis is particularly compelling for species with a continuous distribution and relationship among individuals driven by isolation-by-distance^37^. SNPs with SPA scores above the 99.9^th^ percentile were identified as outliers.

### Environmental Association Analysis

Mixed-model association as implemented in the Factored Spectrally Transformed Linear Mixed Models (FaST-LMM)^57^ was used to test for associations between individual SNPs and bioclimatic and biophysical variables. The following models were explored: K, Q + K, P + K and L + K. The Q + K model was fitted using the equation **y = Xβ + Cγ + Zu + e**, where **y** is a vector of environmental variable; **β** is a vector of fixed marker effects; **γ** is a vector of subpopulation effects; **u** is a vector of polygenic effects caused by relatedness, i.e., ${\bf{u}} \sim MVN(0,\,{\bf{K}}{\sigma }_{u}^{2})$; **e** is a vector of residuals, i.e., ${\bf{e}} \sim MVN(0,{\bf{I}}{\sigma }_{e}^{2})$; **X** is a marker matrix; **C** is an incidence matrix containing membership proportions to each of the three genetic clusters identified by the *fast*STRUCTURE analysis; **Z** is the corresponding design matrix for **u**; and **K** is the realized relationship matrix estimated internally in the FaST-LMM. The K model was the same with the Q + K model except that the term **Cγ** was removed in the model. In the P + K, the incidence matrix C of the Q + K model was replaced with a matrix that contained the first three PCs identified from PCA. In the L + K model, the incidence matrix C of the Q + K model was replaced with a matrix that contained the latitude information corresponding to each accession’s collection site.

A comparison-wise error rate of P < 0.0000143 was used to control the experiment-wise error rate determined by calculating the effective number of SNPs tested^58^. Multiple linear regression was used to estimate the proportion of phenotypic variance accounted for by significant SNPs after accounting for population structure effects.

### Haplotype Analysis

Haplotype blocks were constructed using the four gamete method (4gamete)^59^ implemented in the software Haploview^60^. The 4gamete method creates block boundaries where there is evidence of recombination between adjacent SNPs based on the presence of all four gametic types. A cutoff of 1% was used, meaning that if addition of a SNP to a block resulted in a recombinant allele at a frequency exceeding 1%, then that SNP was not included in the block.

### Candidate Gene Annotations and Enrichment Analysis

SNPs identified as outliers through the environmental association mapping, SPA, or *F*_ST_ approaches were examined for functional annotation using SoyBase (www.soybase.org). A sliding window-approach (e.g., 50 kb) was used to search for functional genes implemented in bedtools^61^. The prediction of candidate genes was based on (a) genes of known function in soybean related to the trait under study, and (b) genes with function-known sequence homologs in *Arabidopsis* related to the trait. For each significant SNP, we collected additional information on genic context, nearby annotated genes, and the inferred Arabidopsis ortholog (TAIR10 best hit provided by Soybase). We performed enrichment analysis to determine if euchromatin, 3′ UTR, 5′ UTR, coding sequence (CDS), and intronic regions were over or under represented among outliers. Significance of enrichment was assessed by creating a 99% confidence interval around the proportion of SNPs that were found in each category as calculated by bootstrap sampling the number of SNPs in each category 1000 times.

## Electronic supplementary material

Supplementary Information

Supplementary Table 1

Supplementary Table 2

Supplementary Table 3

Supplementary Table 4

Supplementary Table 5

Supplementary Table 6

Supplementary Table 7

Supplementary Table 8

Supplementary Table 9

## References

[1] Food and Agriculture Organization. FAOSTAT: Food and Agriculture Data (2014).

[2] United States Department of Agriculture National Agriculture Statistics Service. In *Agricultural Statistics 2007* 514 (United States Government Printing Office, Washington, 2007).

[3] Chadd, S. A., Davies, W. P. & Koivisto, J. M. Practical production of protein for food animals. Proceedings of Protein Sources for the Animal Feed Industry. (*FAO Animal Production and Health*) Available at http://www.fao.org/docrep/007/y5019e/y5019e07.htm (2004).

[4] L’Hocine L, Boye JI (2007). Allergenicity of soybean: new developments in identification of allergenic proteins, cross-reactivities and hypoallergenization technologies. Crit. Rev. Food Sci. Nutr..

[5] Carter, T. E., Nelson, R. L., Sneller, C. H. & Cui, Z. Genetic Diversity in Soybean. Soybeans: Improvement, Production, and uses. *(American Society of Agronomy Monograph Series)*, 303–416 (2004).

[6] Mishra, S. K. & Verma, V. D. In *The Soybean: Botany, Production and* Uses (ed Singh. G.) 74–75 (CAB International, British Library, London, UK, 2010).

[7] Li ZL, Nelson RL (2001). Genetic diversity among soybean accessions from three countries measured by RAPDs. Crop Sci..

[8] Nelson RL (2011). Managing self-pollinated germplasm collections to maximize utilization. Plant Genet..

[9] Cao D (2017). Molecular mechanisms of flowering under long days and stem growth habit in soybean. J. Exp. Bot..

[10] Lu S (2017). Natural variation at the soybean J locus improves adaptation to the tropics and enhances yield. Nat. Genet..

[11] Pan WJ (2017). Soybean NIMA-Related Kinase1 Promotes Plant Growth and Improves Salt and Cold Tolerance. Plant Cell Physiol..

[12] Valliyodan B (2017). Genetic diversity and genomic strategies for improving drought and waterlogging tolerance in soybeans. J. Exp. Bot..

[13] Zhou T (2016). Genotypic Differences in Phosphorus Efficiency and the Performance of Physiological Characteristics in Response to Low Phosphorus Stress of Soybean in Southwest of China. Front. Plant. Sci..

[14] Patil G (2016). Genomic-assisted haplotype analysis and the development of high-throughput SNP markers for salinity tolerance in soybean. Sci. Rep..

[15] Rellstab C, Gugerli F, Eckert AJ, Hancock AM, Holderegger R (2015). A practical guide to environmental association analysis in landscape genomics. Mol. Ecol..

[16] Bragg JG, Supple MA, Andrew RL, Borevitz JO (2015). Genomic variation across landscapes: insights and applications. New Phytol..

[17] Hancock AM (2011). Adaptation to climate across the Arabidopsis thaliana genome. Science.

[18] Fournier-Level A (2011). A map of local adaptation in Arabidopsis thaliana. Science.

[19] Lasky JR (2015). Genome-environment associations in sorghum landraces predict adaptive traits. *Sci*. Adv..

[20] Romero Navarro JA (2017). A study of allelic diversity underlying flowering-time adaptation in maize landraces. Nat. Genet..

[21] Russell J (2016). Exome sequencing of geographically diverse barley landraces and wild relatives gives insights into environmental adaptation. Nat. Genet..

[22] Anderson JE, Kono TJ, Stupar RM, Kantar MB, Morrell PL (2016). Environmental Association Analyses Identify Candidates for Abiotic Stress Tolerance in Glycine soja, the Wild Progenitor of Cultivated Soybeans. G3 (Bethesda).

[23] Song Q (2013). Development and evaluation of SoySNP50K, a high-density genotyping array for soybean. PLoS One.

[24] Song Q (2015). Fingerprinting Soybean Germplasm and Its Utility in Genomic Research. G3 (Bethesda).

[25] Pickrell JK, Pritchard JK (2012). Inference of population splits and mixtures from genome-wide allele frequency data. PLoS Genet..

[26] Kuzmin YV (2013). Long-distance obsidian transport in prehistoric Northeast Asia. Bulletin of the Indo-Pacific Prehistory Association.

[27] Rhee SN, Aikens CM, Ch‘oe SN, No HC (2007). Korean Contributions to Agriculture, Technology, and State Formation in Japan: Archaeology and History of an Epochal Thousand Years, 400 BC–AD 600. Asian Perpectives.

[28] Hymowitz T (1970). On the domestication of the soybean. Econ. Bot..

[29] Peres-Neto PR, Legendre P, Dray S, Borcard D (2006). Variation partitioning of species data matrices: estimation and comparison of fractions. Ecology.

[30] Qin F (2008). Arabidopsis DREB2A-interacting proteins function as RING E3 ligases and negatively regulate plant drought stress-responsive gene expression. Plant Cell.

[31] Yuen CY, Sedbrook JC, Perrin RM, Carroll KL, Masson PH (2005). Loss-of-function mutations of ROOT HAIR DEFECTIVE3 suppress root waving, skewing, and epidermal cell file rotation in Arabidopsis. Plant Physiol..

[32] Vuong TD, Sleper DA, Shannon JG, Nguyen HT (2011). Confirmation of quantitative trait loci for resistance to multiple-HG types of soybean cyst nematode (*Heterodera glycines* Ichinohe). Euphytica.

[33] Orf JH, Chase K, Jarvik T, Mansur LM, Cregan PB (1999). Genetics of soybean agronomic traits: I. comparison of three related recombinant inbred populations. Crop Sci..

[34] Zhang WK (2004). QTL mapping of ten agronomic traits on the soybean (Glycine max L. Merr.) genetic map and their association with EST markers. Theor. Appl. Genet..

[35] Todd JJ, Vodkin LO (1996). Duplications That Suppress and Deletions That Restore Expression from a Chalcone Synthase Multigene Family. Plant Cell.

[36] Tuteja JH, Zabala G, Varala K, Hudson M, Vodkin LO (2009). Endogenous, tissue-specific short interfering RNAs silence the chalcone synthase gene family in glycine max seed coats. Plant Cell.

[37] Yang WY, Novembre J, Eskin E, Halperin E (2012). A model-based approach for analysis of spatial structure in genetic data. Nat. Genet..

[38] Liu B (2010). The soybean stem growth habit gene Dt1 is an ortholog of Arabidopsis TERMINAL FLOWER1. Plant Physiol..

[39] Funatsuki H (2014). Molecular basis of a shattering resistance boosting global dissemination of soybean. Proc. Natl. Acad. Sci. USA.

[40] Kilgore-Norquest L, Sneller CH (2000). Effect of Stem Termination on Soybean Traits in Southern U.S. Production Systems. Crop Sci..

[41] Beaver JS, Johnson RR (1981). Yield Stability of Determinate and Indeterminate Soybeans Adapted to the Northern United States. Crop Sci..

[42] de Silva K, Laska B, Brown C, Sederoff HW, Khodakovskaya M (2011). Arabidopsis thaliana calcium-dependent lipid-binding protein (AtCLB): a novel repressor of abiotic stress response. J. Exp. Bot..

[43] Moran Lauter, A. N. *et al*. Identification of candidate genes involved in early iron deficiency chlorosis signaling in soybean (Glycine max) roots and leaves. *BMC Genomics* **15**, 702-164-15-702 (2014). 10.1186/1471-2164-15-702 PMC4161901 25149281

[44] van Damme M, Huibers RP, Elberse J, Van den Ackerveken G (2008). Arabidopsis DMR6 encodes a putative 2OG-Fe(II) oxygenase that is defense-associated but required for susceptibility to downy mildew. Plant J..

[45] Pandey, N. *et al*. CAMTA 1 regulates drought responses in Arabidopsis thaliana. *BMC Genomics* **14**, 216-2164-14-216 (2013). 10.1186/1471-2164-14-216 PMC3621073 23547968

[46] Yan A, Pan J, An L, Gan Y, Feng H (2012). The responses of trichome mutants to enhanced ultraviolet-B radiation in Arabidopsis thaliana. J. Photochem. Photobiol. B..

[47] Specht, J. E. *et al*. In *Yield Gains in* Major US *Field Crops CSSA. Special Publication 33* (eds Smith, S., Diers, B., Specht, J. & Carver, B.) 311–356 (American Society of Agronomy; Crop Science Society of America; Soil Science Society of America, USA, 2014).

[48] Mourtzinis S (2015). Climate-induced reduction in US-wide soybean yields underpinned by region- and in-season-specific responses. Nat. Plants.

[49] Bandillo N (2015). A population structure and genome-wide association analysis on the USDA soybean germplasm collection. Plant Genome.

[50] Hijmans RJ, Cameron SE, Parra JL, Jones PG, Jarvis A (2005). Very high resolution interpolated climate surfaces for global land areas. Int. J. Climatol..

[51] Hengl T (2014). SoilGrids1km–global soil information based on automated mapping. PLoS One.

[52] Raj A, Stephens M, Pritchard JK (2014). fastSTRUCTURE: variational inference of population structure in large SNP data sets. Genetics.

[53] Purcell S (2007). PLINK: A tool set for whole-genome association and population-based linkage analyses. Am. J. Hum. Genet..

[54] Loecher M, Ropkins K (2015). RgoogleMaps and loa: Unleashing R Graphics Power on Map Tiles. J. Stat. Softw.

[55] Weir BS, Cockerham CC (1984). Estimating F-Statistics for the Analysis of Population Structure. Evolution.

[56] Goudet J (2005). HIERFSTAT, a package for R to compute and test hierarchical F-statistics. Mol. Ecol. Notes.

[57] Lippert C (2011). FaST linear mixed models for genome-wide association studies. Nature Methods.

[58] Li J, Ji L (2005). Adjusting multiple testing in multilocus analyses using the eigenvalues of a correlation matrix. Heredity (Edinb).

[59] Hudson RR, Kaplan NL (1985). Statistical properties of the number of recombination events in the history of a sample of DNA sequences. Genetics.

[60] Barrett JC, Fry B, Maller J, Daly MJ (2005). Haploview: analysis and visualization of LD and haplotype maps. Bioinformatics.

[61] Quinlan AR, Hall IM (2010). BEDTools: a flexible suite of utilities for comparing genomic features. Bioinformatics.

