# Supplementary material for: Dissecting the Genetic Basis of Local Adaptation in Soybean
Source: Sci Rep. 2017 Dec 8;7:17195. doi: 10.1038/s41598-017-17342-w (PMC5722827; doi:10.1038/s41598-017-17342-w)

Supplementary Information

Dissecting the Genetic Basis of Local Adaptation in Soybean

Nonoy B. Bandillo[1], Justin E. Anderson[2], Michael B. Kantar[3], Robert M. Stupar[4], James E. Specht[1], George L. Graef[1] & Aaron J. Lorenz[4]

[1] Dept. of Agronomy & Horticulture, University of Nebraska-Lincoln, Keim Hall Lincoln, NE 68583-0915. [2] Dept. of Molecular Genetics and Physiology of Plants, Ruhr University Bochum, Universitätsstraße 150, Bochum, Germany 40211. [3] Dept. of Tropical Plant and Soil Sciences, University of Hawaii, Manoa, Honolulu, HI, 96822. [4]Dept. of Agronomy and Plant Genetics, University of Minnesota, St. Paul, MN 55108-6026.

Correspondence should be addressed to Aaron Lorenz. (lore0149@umn.edu)

Supplementary Figure Captions

Supplementary Figure 1. Standardized distributions of biophysical and bioclimatic variables (green=spatial variables; brown=soil variables; blue= precipitation variables; yellow=temperature variables).

Supplementary Figure 2. Environmental variability among subpopulations defined by *fast*STRUCTURE for **(a)** selected temperature variables, **(b)** selected precipitation variables, and **(c)** selected soil variables.

Supplementary Figure 3. Monthly series analysis for **(a)** mean precipitation and **(b)** maximum temperatures during the soybean growing season (March-November) for each of the three subpopulations inferred from *fastSTRUCTURE.* SP1 (green) is predominated by accessions collected from Korea; SP2 (red) is predominated by accessions collected from China; SP3 (blue) is predominated by accessions from Japan.

Supplementary Figure 4. Pearson correlation between biophysical and bioclimatic variables. Blue indicates a high positive correlation, white indicates a correlation near zero, and red indicates a high negative correlation.

Supplementary Figure 5. Principal component analysis of environmental variables collected based on geographic coordinates of 3,012 landrace *G. max* accessions. **(a)** The first three PCs were used to infer relationship among variables. **(b)** The first two PCs were used to infer relationship among 3,012 landrace *G. max* accessions.

Supplementary Figure 6. Manhattan and quantile-quantile (QQ) plots generated from environmental association analysis using four linear mixed models: **(a)** K model, **(b)** Q+K model **(c)** P+K model, and **(d)** L+K model. The QQ plots are displayed to compare the distribution of observed p-values to the expected distribution under the null hypothesis of no association. The level of significance and the number of associated signals was substantially reduced using the L+K model.

Supplementary Figure 7. Spatial ancestry analysis (SPA), F_{ST}, and environmental associations across the 20 chromosomes of the *Glycine max* genome. The gray solid vertical line denotes the position of significant SNP associated with environmental variables. A sliding window approach was used to plot F_{ST} and SPA values across the 20 chromosomes. The black dashed line denotes the SPA values across the soybean genome while the remaining solid lines denotes the F_{ST} values within and across countries.

Supplementary Figure 8. Spatial ancestry analysis (SPA), F_{ST}, and environmental associations identified on chromosome 19 of the *Glycine max* genome. **(a)** Environmental association for Mean Diurnal Range identified significant SNP associations that co-localized with *Dt1*, a stem termination gene in soybean. **(b)** SPA and F_{ST} values were plotted based on a sliding-window approach. Notably, highest SPA and F_{ST} values overlapped with significant associations between 44533069 – 45292930 Bp that co-localized with *Dt1*. **(c)** Density plot of allele frequency distribution for Mean Diurnal Range**. (d)** Geographic location of individuals with the “A” allele (gray) or “G” allele (gold) with jitter added to show overlapping samples. Only individuals with

ancestry >80% based on *fastSTRUCTURE* results was plotted. Maps were created with the *RgoogleMaps*[54].

Supplementary Figure 9. Environmental association and within-country selection mapping identified overlapping SNP that co-localized with two widely known genes, stem termination gene (*Dt1*) and shattering resistant gene (*Pdh1*). Examining mean allelic effects of strongest associated SNPs suggested that *Dt1* and *Pdh1* are more important for changing precipitation **(a, d)** than changing temperature **(b, e)** across the soybean growing period. Geographic distribution of individuals carrying respective allele (with jitter added to show overlapping samples) suggests *Dt1* and *Pdh1* loci are important signatures of selection that are likely involved in geographic divergence of soybean. (A=April; O=October). Maps were created with the *RgoogleMaps*[54].

Supplementary Figure 10. Spatial ancestry analysis (SPA), F_{ST}, and significant associations identified on chromosome 9 of the *Glycine max* genome. **(a)** SPA and F_{ST} values were plotted based on a sliding-window approach. The gray solid vertical line denotes the position of significant associations. Notably, highest SPA and F_{ST} values overlapped with significant associations between 1054596 – 1261468 Bp. **(b)** Zoom in on 40 kb region around the significant markers. The Arabidopsis ortholog for the nearest genes, Glyma.09G014700 and Glyma.09G014800, are annotated as Ca^{2+}-dependent lipid-binding (CaLB domain) family protein and oxidoreductase, 2OG-Fe (II) oxygenase family protein, respectively.

Supplementary Figure 11. Spatial ancestry analysis (SPA), F_{ST}, and significant associations identified on chromosome 17 of the *Glycine max* genome. **(a)** SPA and F_{ST} values were plotted based on a sliding-window approach. The gray solid vertical line denotes the position of significant associations. Notably, highest SPA and F_{ST} values overlapped with significant associations between 3857335 – 4466291 Bp. **(b)** Zoom in on 30 kb region around the significant markers. The Arabidopsis ortholog for the nearest genes are annotated as calmodulin-binding and heat-shock transcription factors.

Supplementary Figure 1

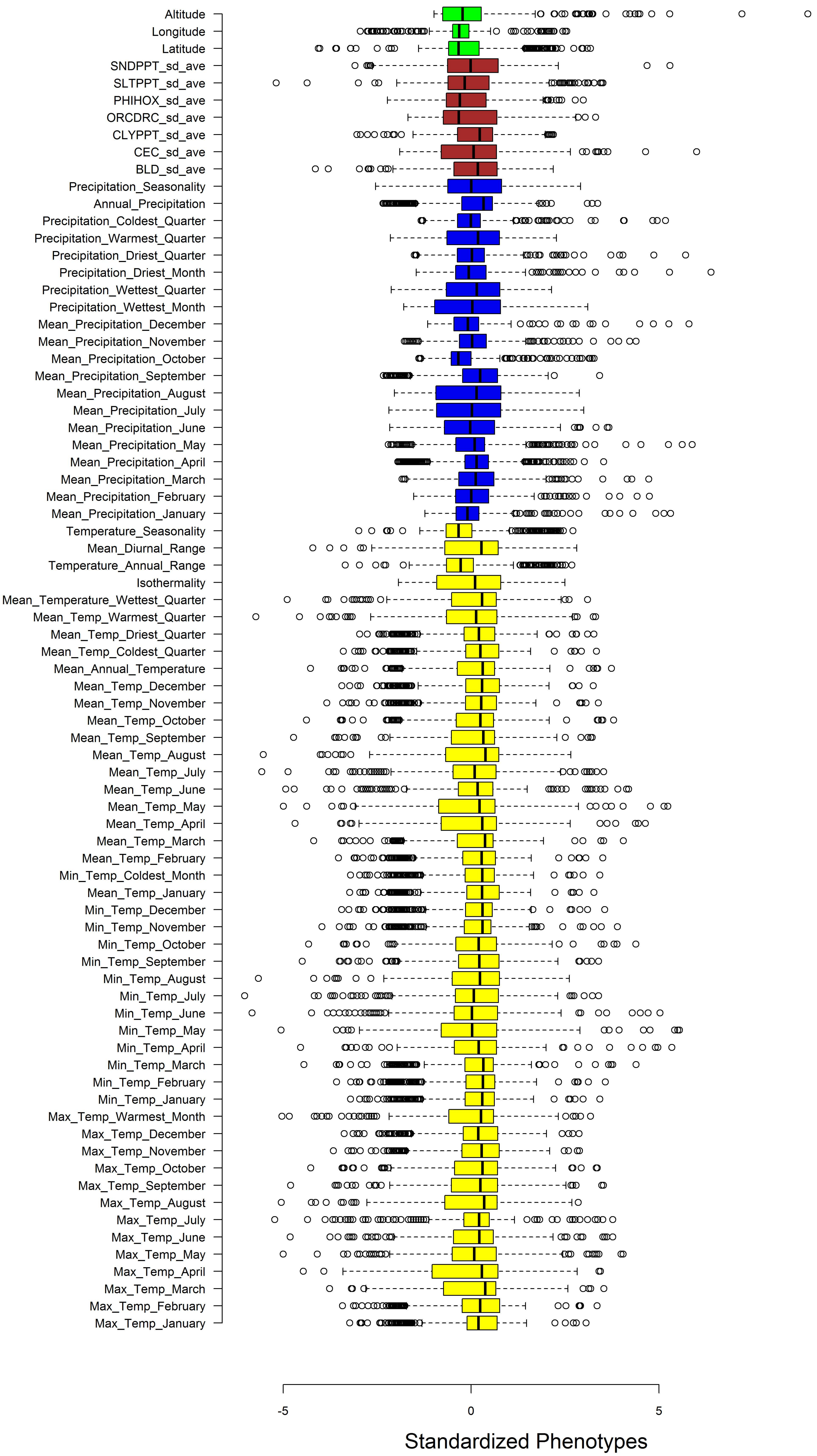

Supplementary Figure 2

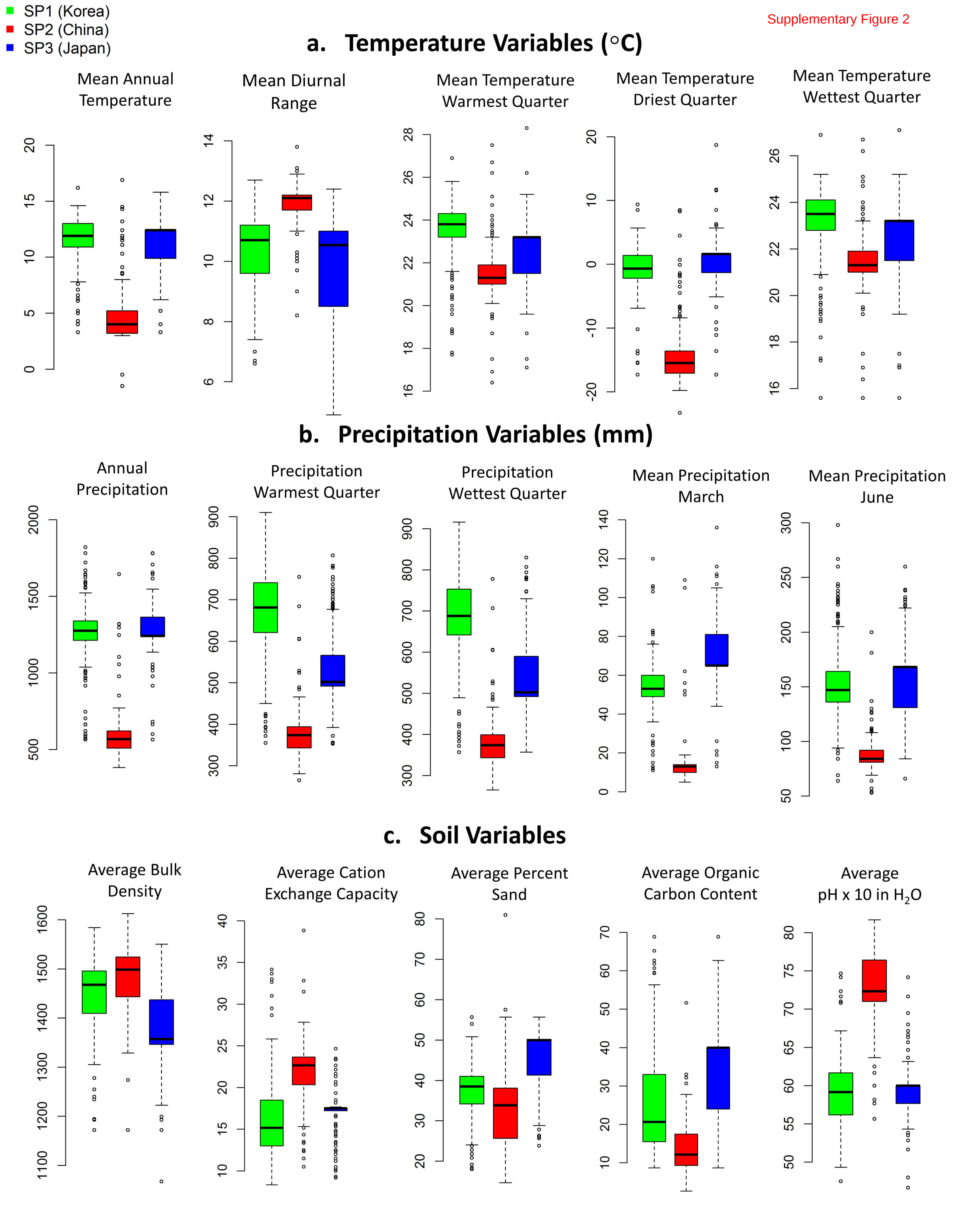

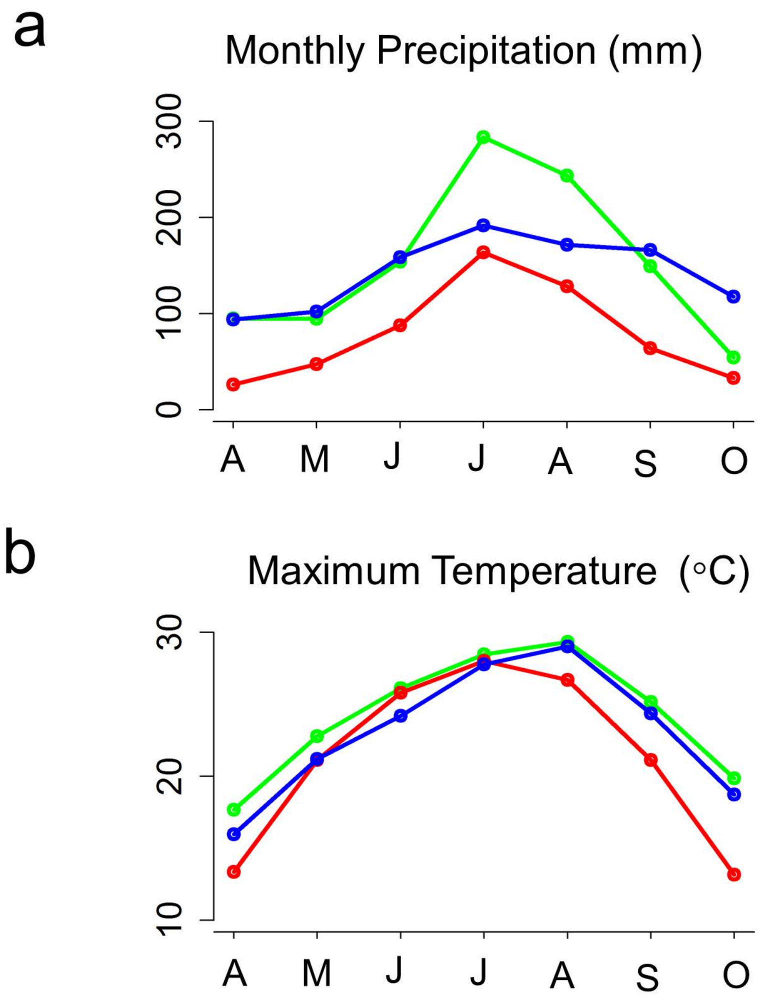
a
Monthly Precipitation (mm)
0
100
200
300
A
M
J
J
A
S
O
b
Maximum Temperature (°C)
10
20
30
A
M
J
J
A
S
O

Supplementary Figure 4

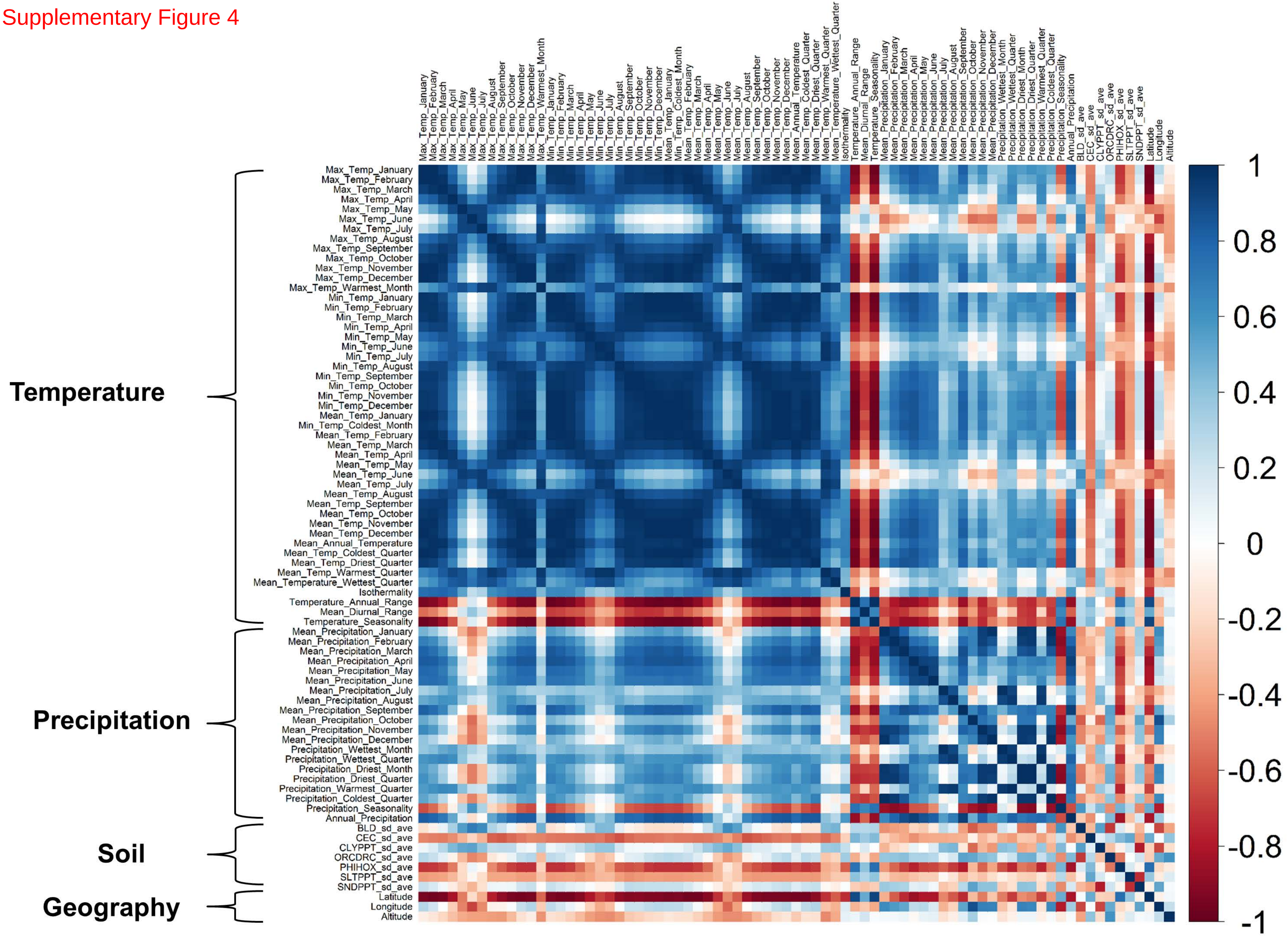

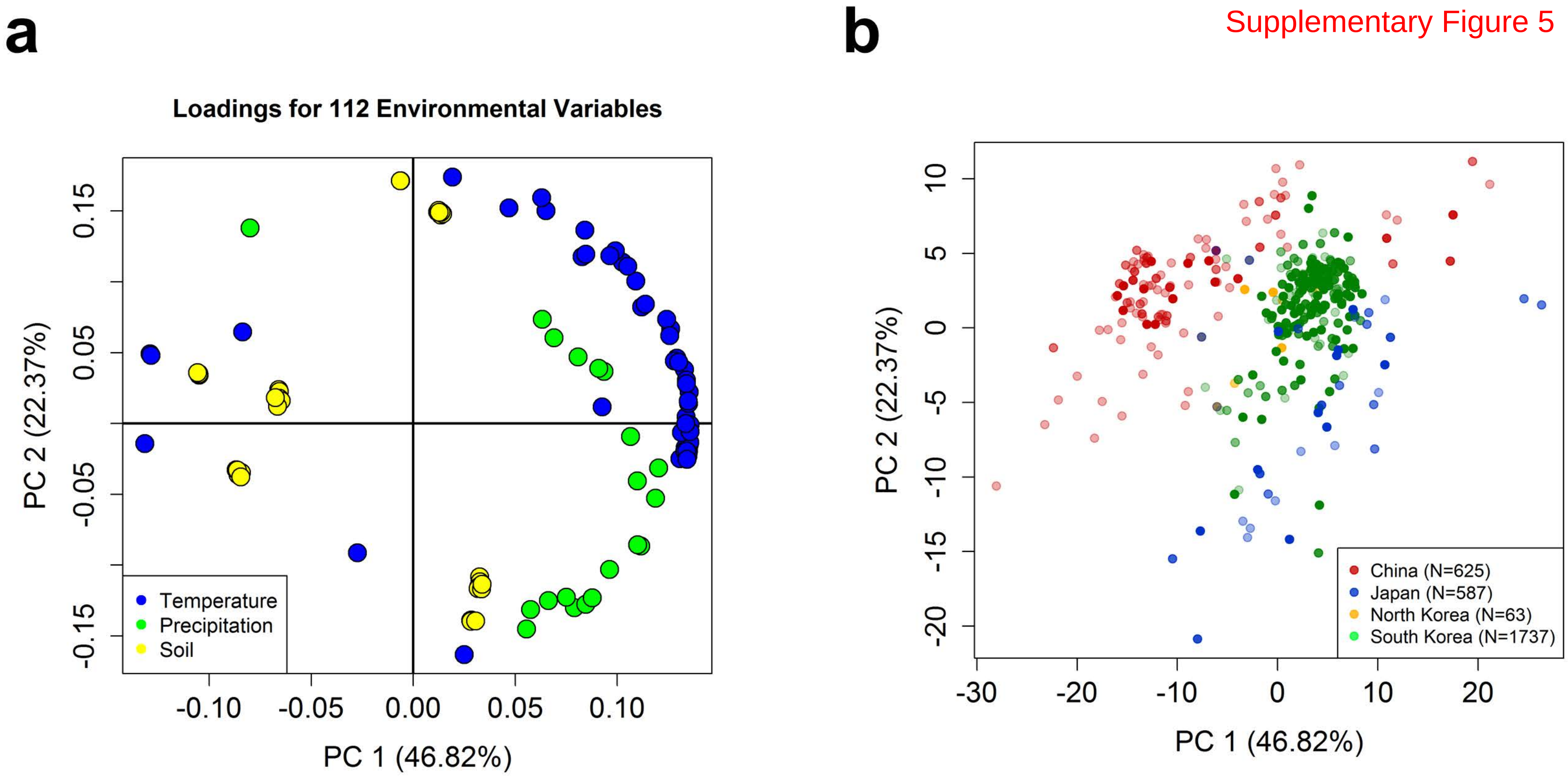
a
Loadings for 112 Environmental Variables
PC 2 (22.37%)
PC 1 (46.82%)
0.15
0.05
-0.05
-0.15
-0.10
-0.05
0.00
0.05
0.10
Temperature
Precipitation
Soil
b
PC 2 (22.37%)
PC 1 (46.82%)
10
5
0
-5
-10
-15
-20
-30
-20
-10
0
10
20
China (N=625)
Japan (N=587)
North Korea (N=63)
South Korea (N=1737)

Supplementary Figure 6

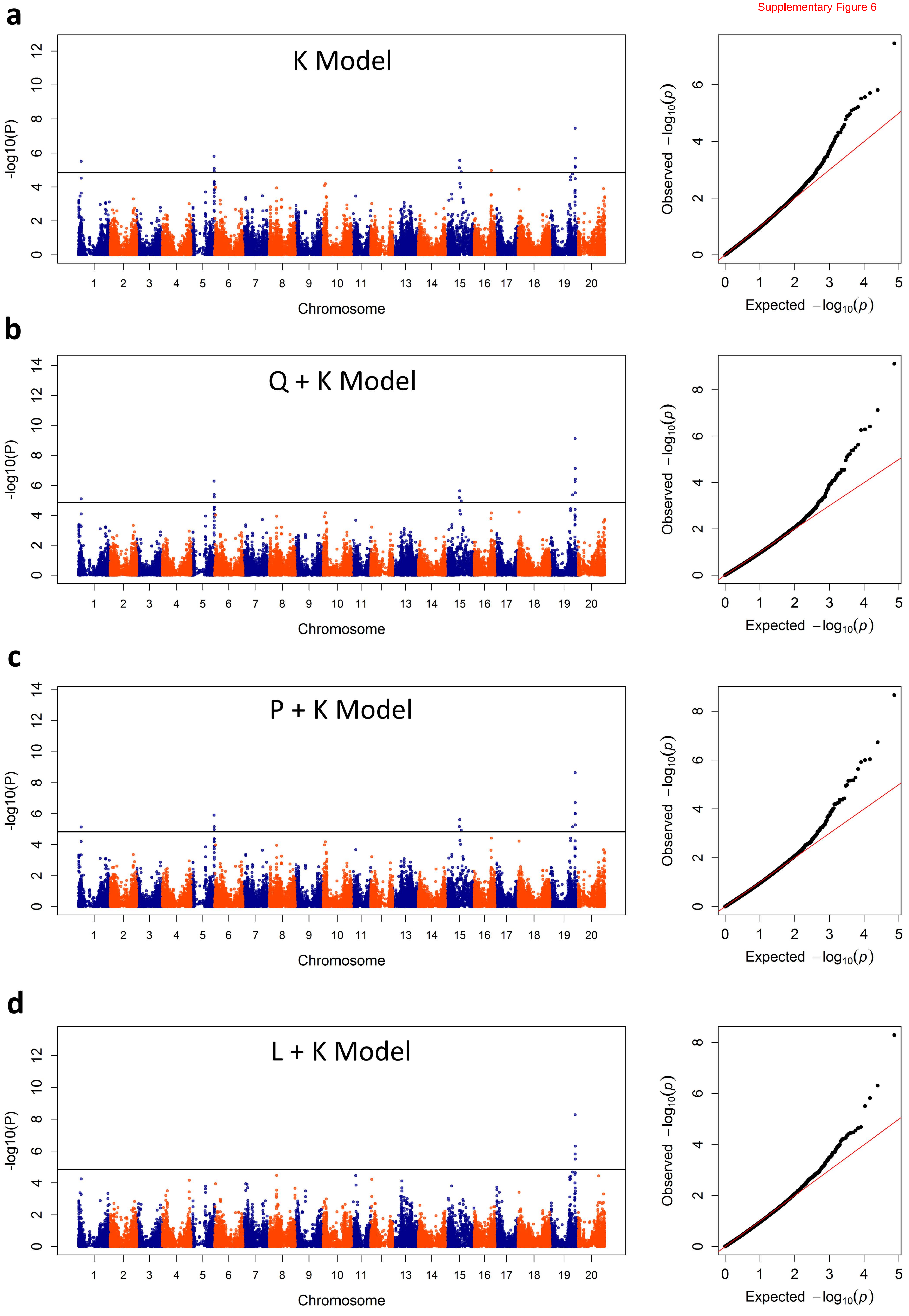

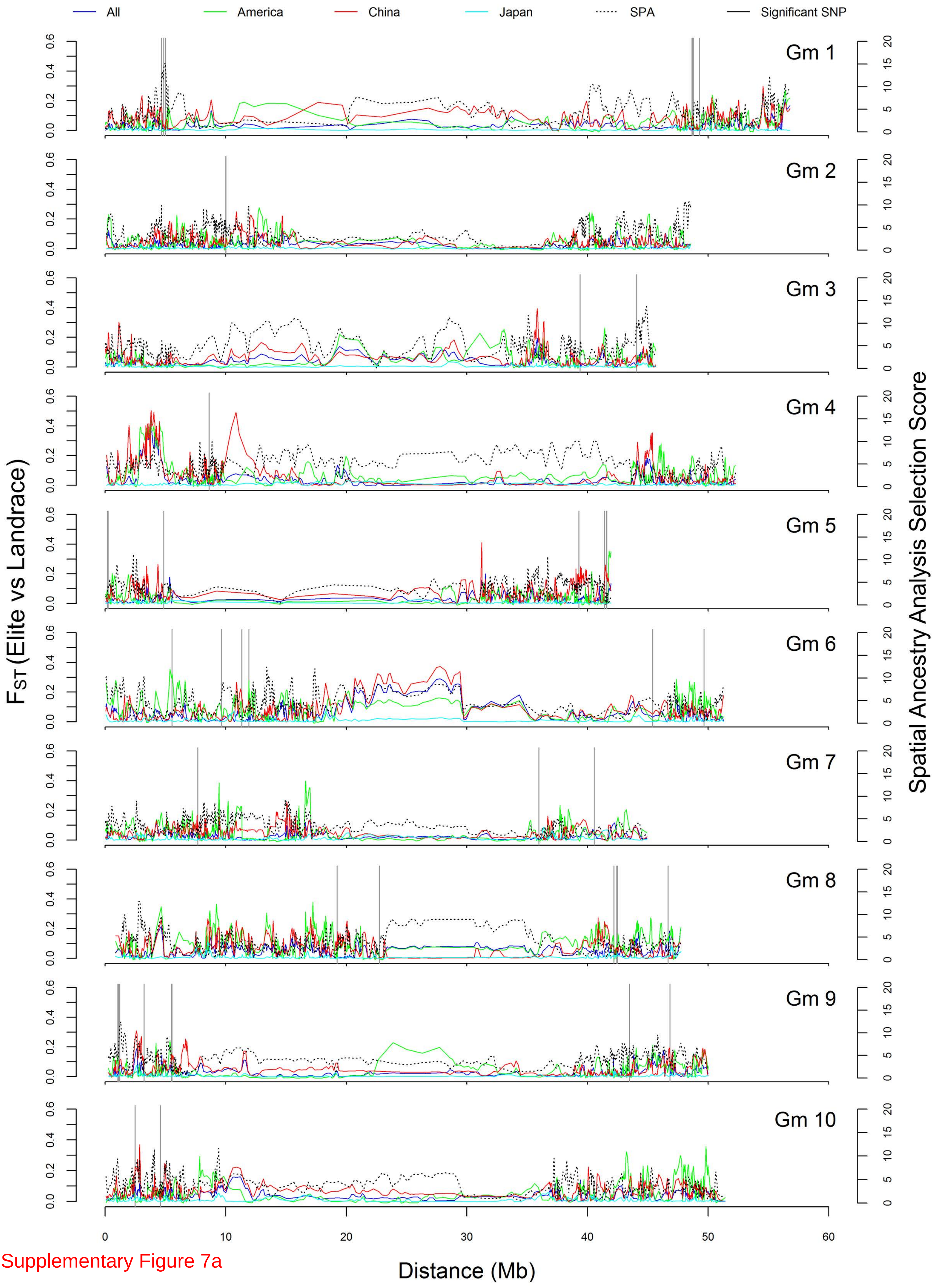

Supplementary Figure 7a

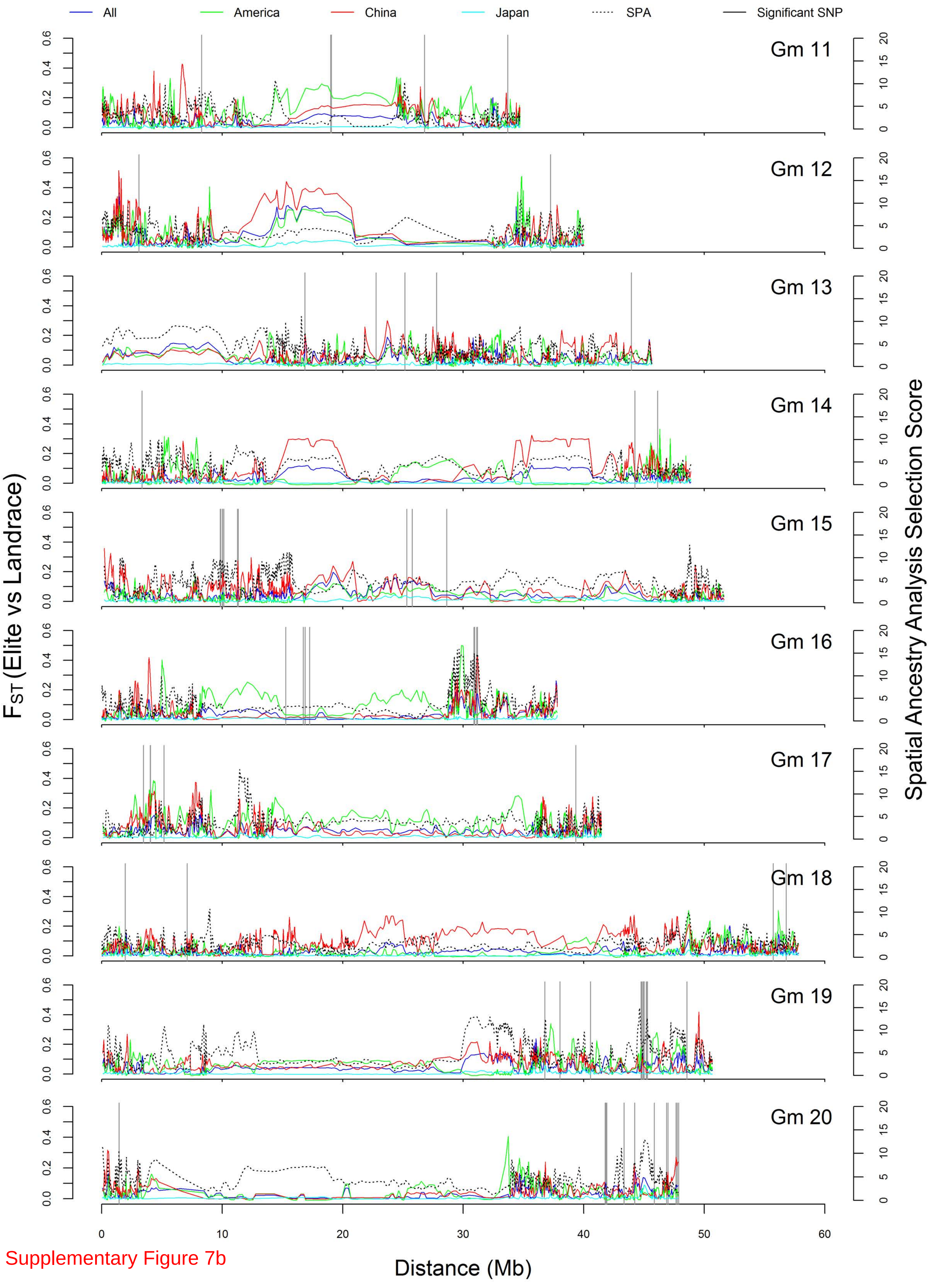

Supplementary Figure 7b

Supplementary Figure 8

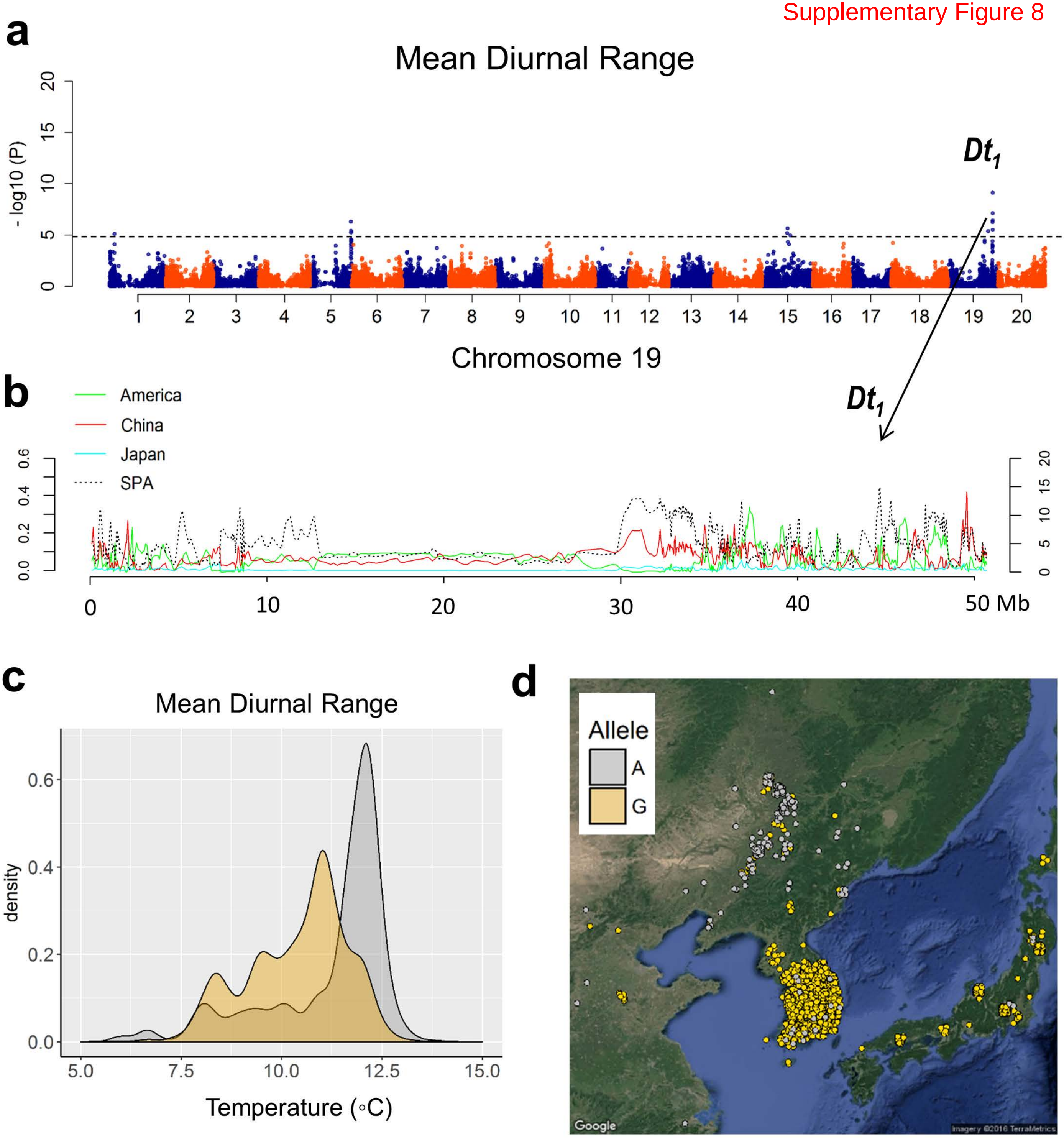

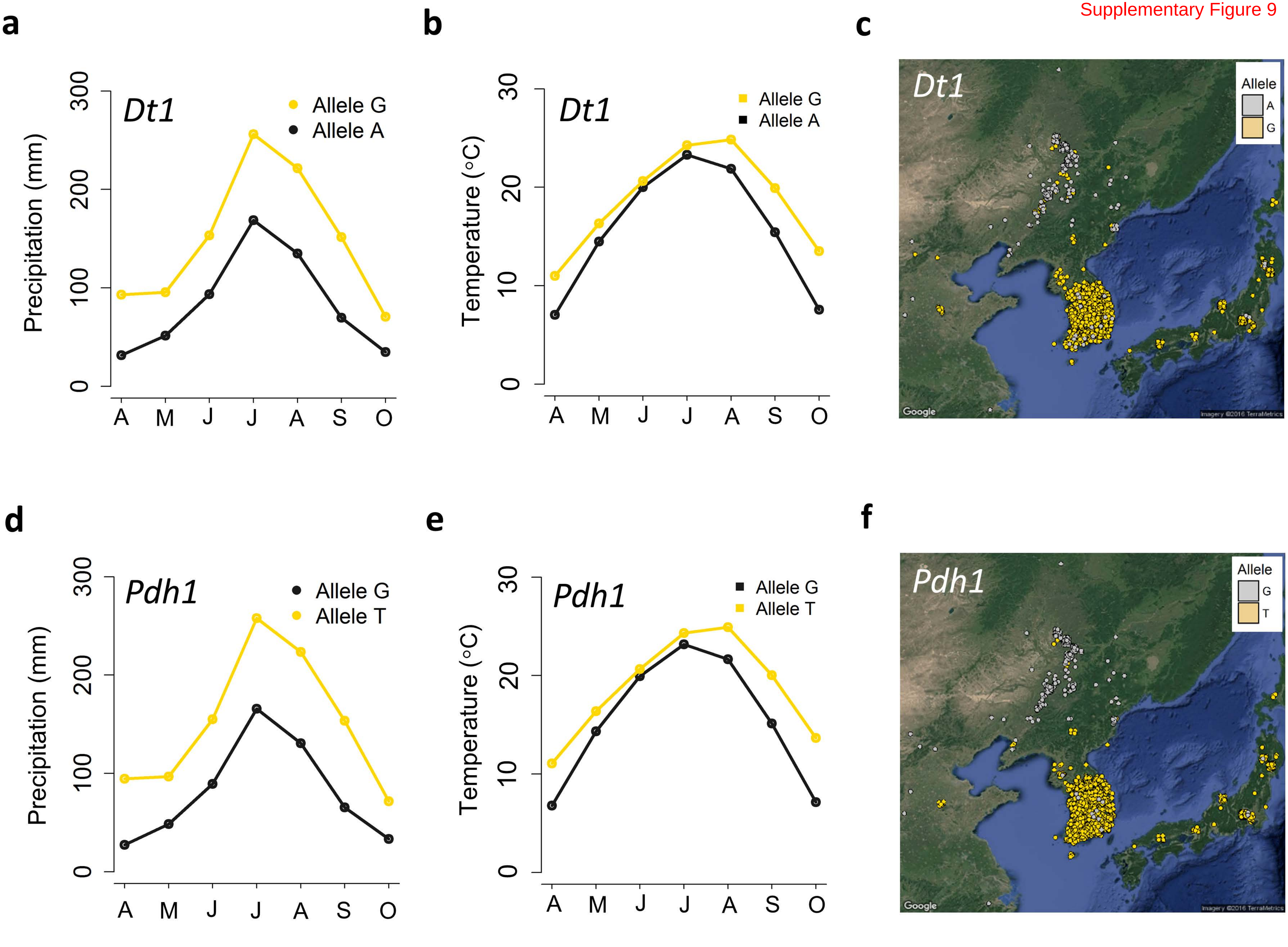
a
Precipitation (mm)
0
100
200
300
Dt1
Allele G
Allele A
A M J J A S O
b
Temperature (°C)
0
10
20
30
Dt1
Allele G
Allele A
A M J J A S O
c
Dt1
Allele
A
G
Google
d
Precipitation (mm)
0
100
200
300
Pdh1
Allele G
Allele T
A M J J A S O
e
Temperature (°C)
0
10
20
30
Pdh1
Allele G
Allele T
A M J J A S O
f
Pdh1
Allele
G
T
Google

Supplementary Figure 10

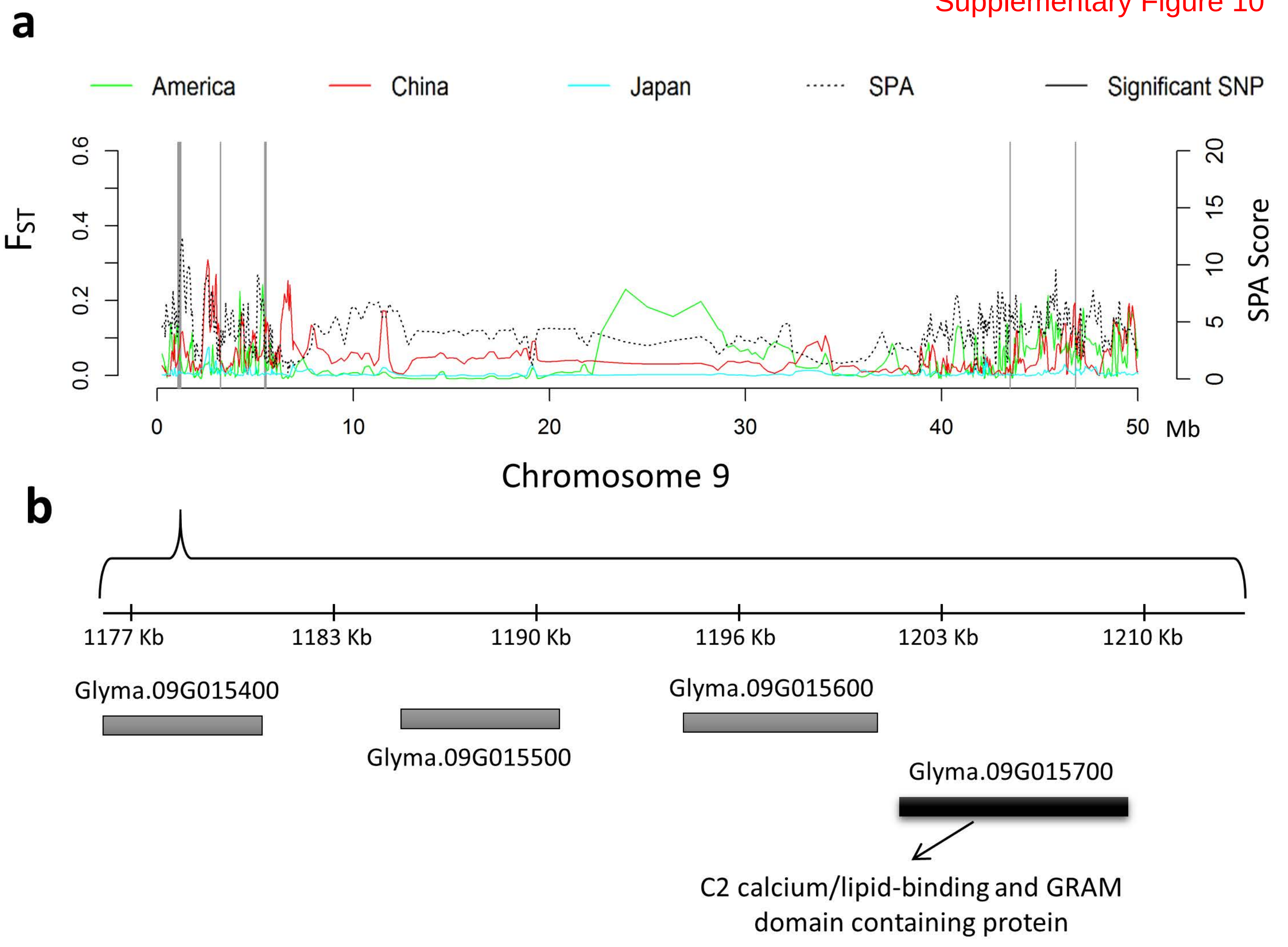

Supplementary Figure 11

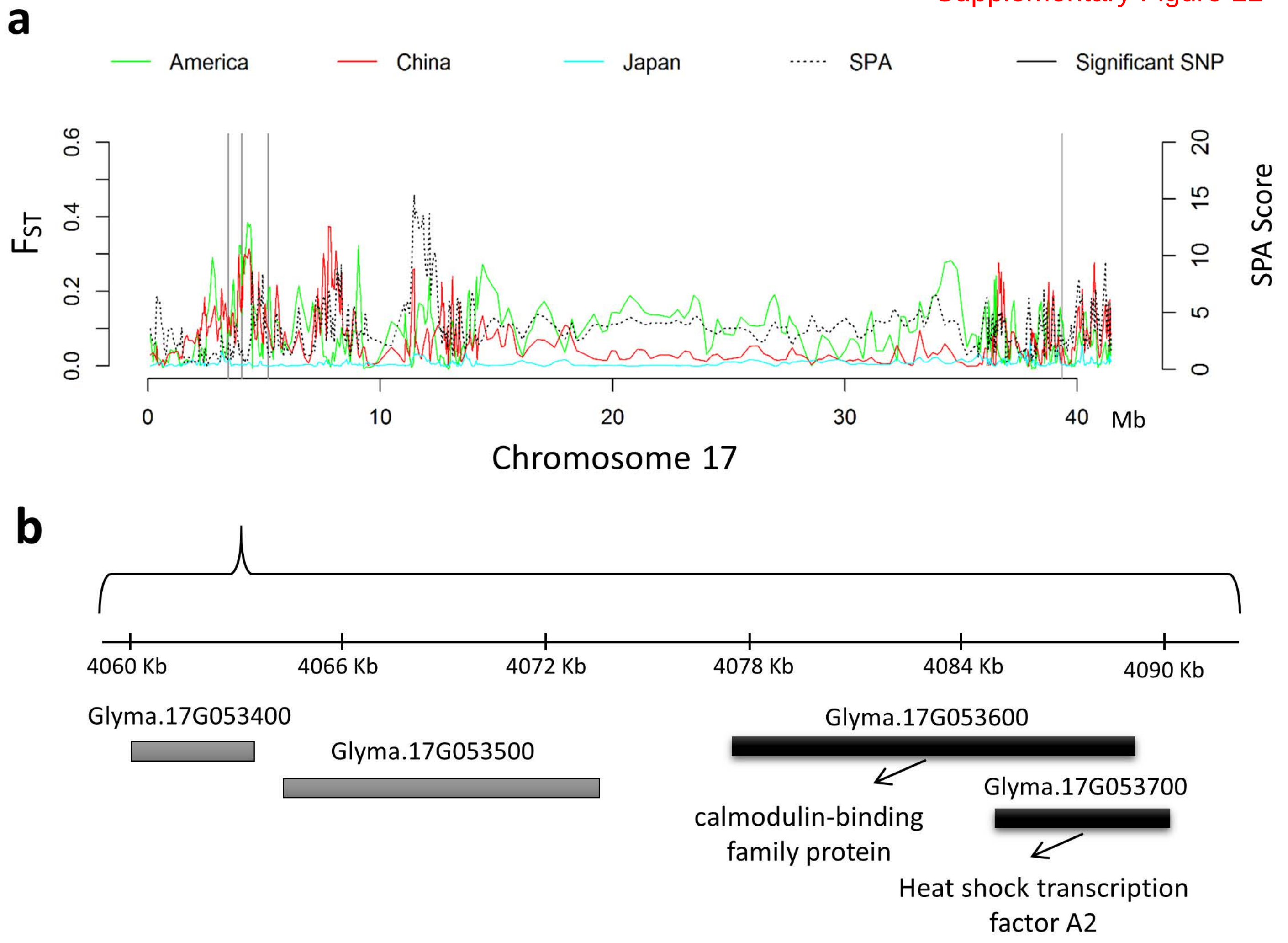

Supplement: Supplementary file 1 — Supplementary Information [file 41598_2017_17342_MOESM1_ESM.pdf]
